# Supplementary material for: Preclinical evaluation of the SARS-CoV-2 Mpro inhibitor RAY1216 shows improved pharmacokinetics compared with nirmatrelvir
Source: Nat Microbiol. 2024 Mar 29;9(4):1075–88. doi: 10.1038/s41564-024-01618-9 (PMC10994847; doi:10.1038/s41564-024-01618-9)
Supplement: Supplementary file 1 — Supplementary Discussion, Figs. 1–13, Tables 1–8 and References. [file 41564_2024_1618_MOESM1_ESM.pdf]

nature microbiology

Article https://doi.org/10.1038/s41564-024-01618-9

Preclinical evaluation of the SARS-CoV-2 M^{pro} inhibitor RAY1216 shows improved pharmacokinetics compared with nirmatrelvir

In the format provided by the authors and unedited

Supplementary Information

Contents

S1 Synthesis and characterization of RAY1216 **S2**
S1.1 Synthesis of RAY1216 . . . . . S2
S1.2 NMR spectra of RAY1216 . . . . . S6
S1.3 X-ray crystal structure of RAY1216 . . . . . S9

S2 Enzyme kinetics **S11**
S2.1 Enzyme activity assay S11
S2.2 Molar response coefficient of fluorescent product S11
S2.3 Determination of the Michaelis constant, K_M S12
S2.4 Inhibition kinetics of PF-07321332 and active-site titration S15
S2.5 Inhibition kinetics of RAY1216 . . . . . S19
S2.6 Inhibition of the P132H mutant S22

S3 M^{pro} crystal structures **S23**
S3.1 Simulated-annealing 2mFo-DFc composite omit map densities S23
S3.2 Comparison of active site structures S24
S3.3 Protein crystallographic refinement statistics data S25

S4 Antiviral activity of RAY1216 **S26**
S4.1 Cytotoxicity of RAY1216 in Vero E6 cells S26
S4.2 Antiviral titres of RAY1216 and PF-07321332 in Vero E6 cells S27

S5 Pharmacokinetics **S28**
S5.1 Pharmacokinetics of RAY1216 in K18-hACE2 transgenic mice S28

Supplementary References **S29**

S1. Synthesis and characterization of RAY1216

S1.1. Synthesis of RAY1216

Step 1: Synthesis of the methyl (S)-2-amino-3-((S)-2-oxopyrrolidin-3-yl)propanoate hydrochloride (RAY1216-2)

To a solution of methyl (S)-2-((tert-butoxycarbonyl)amino)-3-((S)-2-oxopyrrolidin-3-yl)propanoate (RAY1216-1) (500 mg, 1.75 mmol) in ethyl acetate (5 mL) at 20 °C, HCl/EA (10 mL, 4 M) was added. The resulting mixture was stirred for 2 h at 20 °C. Solvent was removed to afford the residue as unpurified RAY1216-2 hydrochloride salt (388 mg, 1.75 mmol, 99% yield). ^{1}H NMR (400 MHz, CD_3OD) δ = 4.28 - 4.20 (m, 1H), 3.91 - 3.81 (m, 3H), 3.45 - 3.35 (m, 2H), 2.86 - 2.74 (m, 1H), 2.48 - 2.36 (m, 1H), 2.29 - 2.19 (m, 1H), 2.02 - 1.94 (m, 1H), 1.93 - 1.80 (m, 1H).

Step 2: Synthesis of ethyl (1S,3aR,6aS)-2-(2-((tert-butoxycarbonyl)amino)-2-cyclohexylacetyl)octahydrocyclopenta-[c]pyrrole-1-carboxylate (RAY1216-4)

To a solution of Boc-L-cyclohexylglycine (1 g, 3.89 mmol) in N,N-dimethylformamide (10 mL), 2-(7-azobenzo-triazole)-N,N-tetramethylurea hexafluorophosphate (1.77 g, 4.66 mmol) was added. The resulting mixture was stirred for 0.5 h, to which diisopropylethylamine (1.26 g, 9.72 mmol) and ethyl (1S,3aR,6aS)-octahydrocyclopenta[c]pyrrole-1-carboxylate (RAY1216-3) hydrochloride salt (1.02 g, 4.66 mmol) were added. The resulting mixture was stirred for 16 h at 20 °C. The reaction mixture was added to methyl tert-butyl ether (50 mL), water (20 mL) and washed with 3% citric acid (20 mL×2) and brine (20 mL). The combined organic phase was dried over anhydrous sodium sulfate. After solvent removal, the residue purified by silica gel column chromatography (petroleum ether : ethyl acetate = 3:1) to afford RAY1216-4 (1.41g, 3.34 mmol, 86% yield). ^{1}H NMR (400 MHz, $CDCl_3$) δ = 5.22 - 5.11 (m, 1H), 4.36 (d, J=3.9 Hz, 1H), 4.27 (dd, J=6.9, 9.3 Hz, 1H), 4.21 - 4.12 (m, 2H), 3.83 (dd, J=7.8, 10.4 Hz, 1H), 3.70 (br dd, J=3.6, 10.4 Hz, 1H), 2.81 - 2.61 (m, 2H), 1.82 - 1.70 (m, 6H), 1.68 - 1.61 (m, 4H), 1.56 - 1.48 (m, 2H), 1.46 - 1.38 (m, 9H), 1.29 - 1.22 (m, 4H), 1.21 - 0.98 (m, 4H). LCMS (m/z) 423.3 $[M+1]^+$.

Step 3: Synthesis of (1S,3aR,6aS)-2-((S)-2-((tert-butoxycarbonyl)amino)-2-cyclohexylacetyl) octahydrocyclopenta-[c]pyrrole-1-carboxylic acid (RAY1216-5)

To a THF (14 mL) solution of RAY1216-4 (1.41g, 3.34 mmol), $LiOH \cdot H_2O$ (280.03 mg, 6.67 mmol) in water (5 mL) was added. The resulting mixture was stirred for 16 h at 20 °C. Citric acid was added to the reaction mixture to 3%. Solvent was removed and residue was extracted with ethyl acetate (50 mL), washed with brine (30 mL). The combined organic phase was dried over anhydrous sodium sulfate. Solvent was removed to afford the residue as unpurified RAY1216-5 (650 mg, 1.65 mmol, 49% yield). ^{1}H NMR (400 MHz, DMSO-d_6) δ = 12.58 - 12.23 (m, 1H), 6.92 - 6.82 (m, 1H), 4.11 - 3.94 (m, 2H), 3.82 - 3.76 (m, 1H), 3.72 - 3.62 (m, 1H), 2.73 - 2.64 (m, 1H), 2.62 - 2.55 (m, 1H), 1.92 - 1.42 (m, 12H), 1.40 - 1.32 (m, 9H), 1.18 - 1.06 (m, 3H), 1.00 - 0.81 (m, 2H). LCMS (m/z) 395.3 $[M+1]^+$.

Step 4: Synthesis of methyl (S)-2-((1S,3aR,6aS)-2-((S)-2-((tert-butoxycarbonyl)amino)-2-cyclohexylacetyl)octahydro-cyclopenta[c]pyrrole-1-carboxamido)-3-((S)-2-oxopyrrolidin-3-yl)propanoate (RAY1216-6)

To a 2-butanone (7 mL) solution of RAY1216-5 (650 mg, 1.65 mmol), 1-hydroxybenzotriazole (222.63 mg, 1.65 mmol), 1-(3-dimethylaminopropyl)-3-ethylcarbodiimide hydrochloride (379.03 mg, 1.98 mmol), diisopropylethy-lamine (638.84 mg, 4.94 mmol) were added. The resulting mixture was stirred for 0.5 h at 20 °C, before RAY1216-2 hydrochloride salt (366.88 mg, 1.65 mmol) was added. The resulting mixture was stirred for 16 h at 20 °C. The re-action mixture was diluted with water (20 mL) and extracted with dichloromethane : methanol (30 mL×2, 10:1), the combined organic phase was washed with 3% citric acid (20 mL× 2), brine (30 mL). The organic phase was dried over anhydrous sodium sulfate. Solvent was removed and the residue was purified by silica gel column chromatography (dichloromethane : methanol = 20:1) to afford RAY1216-6 (834 mg, 1.48 mmol, 89% yield). ^{1}H NMR (400 MHz, $CDCl_3$) δ = 7.49 - 7.42 (m, 1H), 6.23 - 6.05 (m, 1H), 5.28 - 5.17 (m, 1H), 4.64 - 4.51 (m, 1H), 4.43 - 4.24 (m, 2H), 3.92 - 3.81 (m, 1H), 3.78 - 3.70 (m, 3H), 3.39 - 3.27 (m, 2H), 2.94 - 2.75 (m, 2H), 2.57 - 2.36 (m, 2H), 2.24 - 2.07 (m, 1H), 1.94 - 1.50 (m, 14H), 1.49 - 1.41 (m, 9H), 1.27 - 0.95 (m, 6H). LCMS (m/z) 563.4 $[M+1]^+$.

Step 5: Synthesis of tert-butyl ((S)-1-cyclohexyl-2-((1S,3aR,6aS)-1-(((S)-1-hydroxy-3-((S)-2-oxopyrrolidin-3-yl)propan-2-yl)carbamoyl)hexahydrocyclopenta[c]pyrrol-2(1H)-yl)-2-oxoethyl)carbamate (RAY1216-7)

To a THF (31 mL) solution of RAY1216-6 (3.10 g, 5.51 mmol) at 0 °C, lithium borohydride (240.02 mg, 11.02 mmol) was added. Temperature of the mixture was allowed to warm to 20 °C slowly and stirred for 2 h at 20 °C. The mixture was diluted with water (10 mL) and ethyl acetate (20 mL) and stirred for 10 min, white solid was collected as crude target product RAY1216-7 (2.94 g, 5.51 mmol, 99% yield) by filtration. LCMS (m/z) 535.4 $[M+1]^+$.

Step 6: Synthesis of tert-butyl ((S)-1-cyclohexyl-2-oxo-2-((1S,3aR,6aS)-1-(((S)-1-oxo-3-((S)-2-oxopyrrolidin-3-yl)propan-2-yl)carbamoyl)hexahydrocyclopenta[c]pyrrol-2(1H)-yl)ethyl)carbamate (RAY1216-8)

To a DCM (10 mL) solution of compound RAY1216-7 (0.5 g, 935.13 µmol) Dess-Martin periodinane (594.94 mg, 1.40 mmol) was added. The resulting mixture was stirred for 16 h. The mixture was diluted with saturated sodium thiosulfate (15 mL) and saturated sodium bicarbonate solution (15 mL) and stirred for 10 min. The aqueous layer was extracted with DCM (50 mL×2). The combined organic phase was washed with brine (5 mL) and dried over anhydrous sodium sulfate. Solvent was removed and to afford the residue as unpurified RAY1216-8 (436 mg, 818.52 µmol, 87.5% yield). LCMS (m/z) 533.4 $[M+1]^+$.

Step 7: Synthesis of (3S)-3-((1S,3aR,6aS)-2-((S)-2-((tert-butoxycarbonyl)amino)-2-cyclohexylacetyl)octahydrocyclopenta[c]pyrrole-1-carboxamido)-1-(cyclopentylamino)-1-oxo-4-((S)-2-oxopyrrolidin-3-yl)butan-2-yl acetate (RAY1216-9)

To a DCM (10 mL) solution of RAY1216-8 (436 mg, 818.52 µmol), acetic acid (58.98 mg, 982.22 mmol) and cyclopentyl isocyanide (94.44 mg, 982.22 µmol) were added. The resulting mixture was stirred for 2 h at 25 °C. After addition of saturated ammonium chloride solution (10 mL), the reaction mixture was stirred for 10 min and extracted with DCM (20 mL). The combined organic phase was washed with brine (5 mL). The organic phase was dried over anhydrous sodium sulfate, solvent was removed and residue purified by silica gel column chromatography (dichloromethane : methanol = 10:1) to afford RAY1216-9 (396.6 mg, 577.0 µmol, 70.5% yield). LCMS (m/z) 688.4 $[M+1]^+$.

Step 8: Synthesis of tert-butyl ((1S)-1-cyclohexyl-2-((1S,3aR,6aS)-1-(((2S)-4-(cyclopentylamino)-3-hydroxy-4-oxo-1-((S)-2-oxopyrrolidin-3-yl)butan-2-yl)carbamoyl)hexahydrocyclopenta[c]pyrrol-2(1H)-yl)-2-oxoethyl)carbamate (RAY1216-10)

To a MeOH (3 mL) solution of RAY1216-9 (190 mg, 276.22 µmol), K_2CO_3 (95.44 mg, 690.54 µmol) in water (5 mL) was added. The resulting mixture was stirred for 16 h at 20 °C. The reaction mixture was diluted with 3% citric acid and extracted with DCM (40 mL×3), the combined organic phase was washed with brine (30 mL) and dried over anhydrous sodium sulfate. Solvent was removed to afford the residue as unpurified crude RAY1216-10 (170.89 mg, 248.60 µmol, 90.0% yield).

Step 9: Synthesis of tert-butyl ((S)-1-cyclohexyl-2-((1S,3aR,6aS)-1-(((S)-4-(cyclopentylamino)-3,4-dioxo-1-((S)-2-oxopyrrolidin-3-yl)butan-2-yl)carbamoyl)hexahydrocyclopenta[c]pyrrol-2(1H)-yl)-2-oxoethyl)carbamate (RAY1216-11)

To a DCM (10 mL) solution of RAY1216-10 (238.00 mg, 368.52 µmol), Dess-Martin periodinane (203.19 mg, 479.08 µmol) was added. The resulting mixture was stirred for 16 h at 20 °C. The reaction mixture was diluted with saturated sodium thiosulfate (15 mL) and saturated sodium bicarbonate solution (15 mL), extracted with DCM (50 mL×2), and washed with brine (15 mL). The organic phase was dried over anhydrous sodium sulfate to remove solvent. The residue was purified by silica gel column chromatography (dichloromethane : methanol = 20:1) to afford RAY1216-11 (125 mg, 194.16 µmol, 52.6% yield).

Step 10: Synthesis of (1S,3aR,6aS)-2-((S)-2-amino-2-cyclohexylacetyl)-N-((S)-4-(cyclopentylamino)-3,4-dioxo-1-((S)-2-oxopyrrolidin-3-yl)butan-2-yl)octahydrocyclopenta[c]pyrrole-1-carboxamide (RAY1216-12)

To a THF (3 mL) solution of RAY1216-11 (125 mg, 194.16 µmol), HCl/EA (2.91 mL, 4 M) was added. The resulting mixture was stirred for 1 h at 25 °C. Solvent was removed to afford crude RAY1216-12 (93.89 mg, 172.80 µmol, 89.0% yield).

Step 11: Synthesis of (1S,3aR,6aS)-2-((S)-2-cyclohexyl-2-(2,2,2-trifluoroacetamido)acetyl)-N-((S)-4-(cyclopentylamino) -3,4-dioxo-1-((S)-2-oxopyrrolidin-3-yl)butan-2-yl)octahydrocyclopenta[c]pyrrole-1-carboxamide (RAY1216)

To a THF (3 mL) solution of RAY1216-12 (125 mg, 229.91 µmol) at 0 °C, TFAA (193.15 mg, 919.63 µmol) and pyridine (127.30 mg, 1.61 mmol) were added. The resulting mixture was stirred for 16 h at 20 °C. The reaction mixture was diluted with water (20 mL) and extracted with dichloromethane (30 mL×2). The combined organic phase was washed with 3% citric acid (40 mL) and brine (40 mL×2). The organic phase was dried over anhydrous sodium sulfate. Solvent was removed and the residue was purified by HPLC to afford RAY1216 (125 mg, 195.42 µmol, 85.0% yield); mp: 187.6 - 188.5 °C; TLC (CHC_3:MeOH, 20:1 v/v): R_f = 0.49; $[\alpha]_D^{20}$ = -46.7 ° (c=0.97, EtOH).

^{1}H NMR (400 MHz, DMSO-d_6) δ ppm 9.75 d (J=7.5 Hz,1H) 8.64 d (J=7.5 Hz,1H), 8.50 d (J=8.3 Hz,1H), 7.65 s (1H), 5.14 ddd (J=11.5, 8.3, 2.9 Hz,1H), 4.28 t (J=8.6 Hz,1H), 4.20 d (J=4.2 Hz,1H), 4.04 m (1H), 3.76 AABB-d (J=10.2, 7.6, 3.0 Hz,2H), 3.20 - 3.11 m (2H), 2.69 m (1H), 2.55 m (1H), 2.50 m (1H), 2.20 - 1.69 m (2H), 1.88 - 1.60 m (2H), 1.83 m (1H), 1.80 -1.40 m (6H), 1.80 - 1.50 m (4H), 1.80 - 1.40 m (4H), 1.79 - 0.95 m (4H), 1.65 - 1.13 m (6H).

^{13}C NMR (100 MHz, DMSO-d_6) δ ppm 115.9, 156.5, 56.1, 38.7, 28.2, 28.6, 25.4, 25.6, 25.8, 168.6, 53.5, 42.7, 24.6, 31.2, 31.66, 47.5, 65.5, 171.9, 51.8, 31.8, 37.5, 178.4, 39.5, 27.5, 197.2, 161.0, 50.4, 23.6, 31.64, 31.68.IR(Nuiol): 3269.82, 2932.90,2867.26, 1673.07, 1632.70, 1526.34, 1448.84, 714.51 cm^{-1}; UV/Vis: max 194 nm; HRMS(m/z): $[M+H]^+$calcd. for $C_{31}H_{45}F_3N_5O_6$, 640.3322; found, 640.3319.

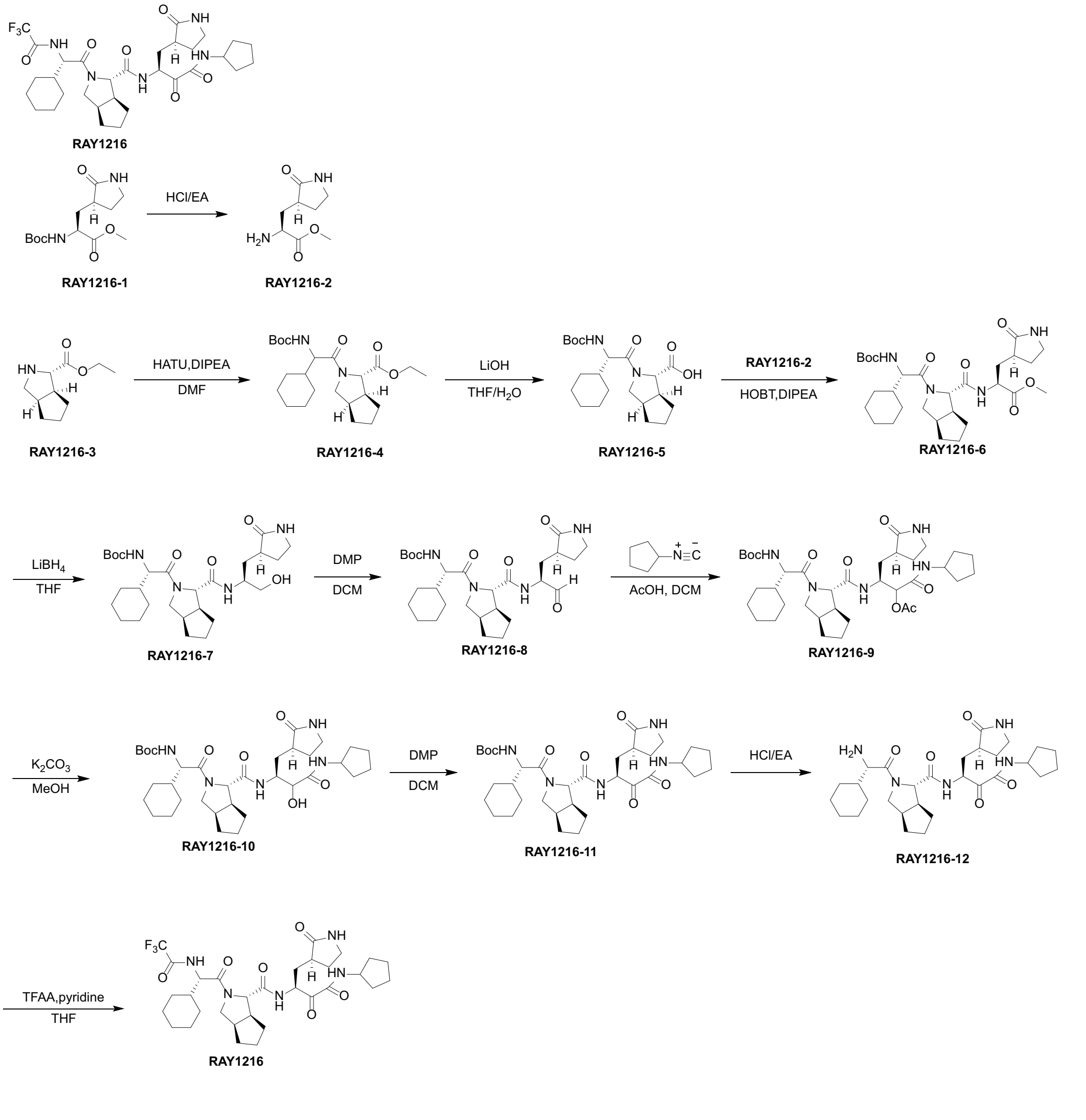

Supplementary Fig. 1 | Synthesis of RAY1216.

S1.2. NMR spectra of RAY1216

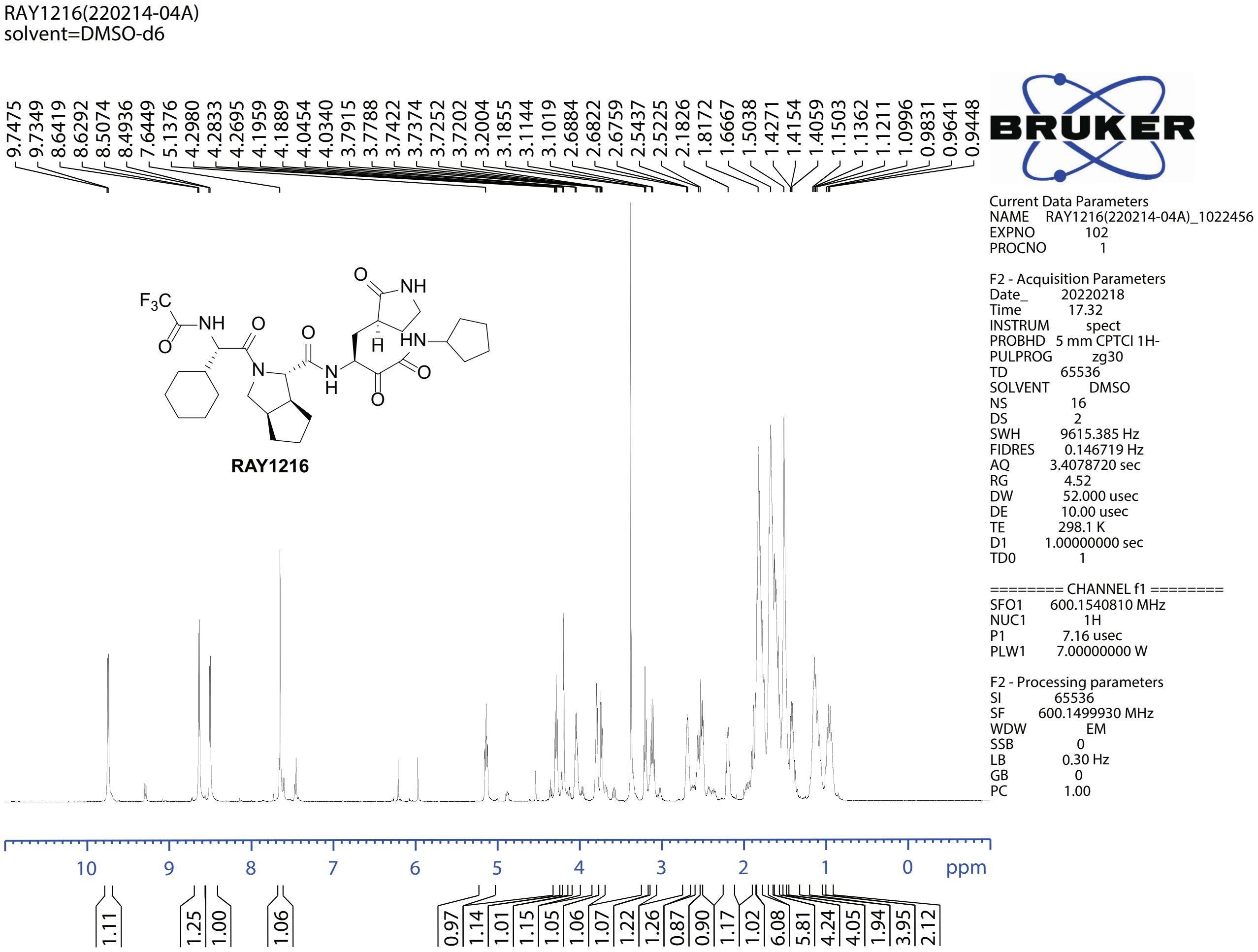

Supplementary Fig. 2 | ^{1}H NMR spectrum of RAY1216 in DMSO-d$_6$.

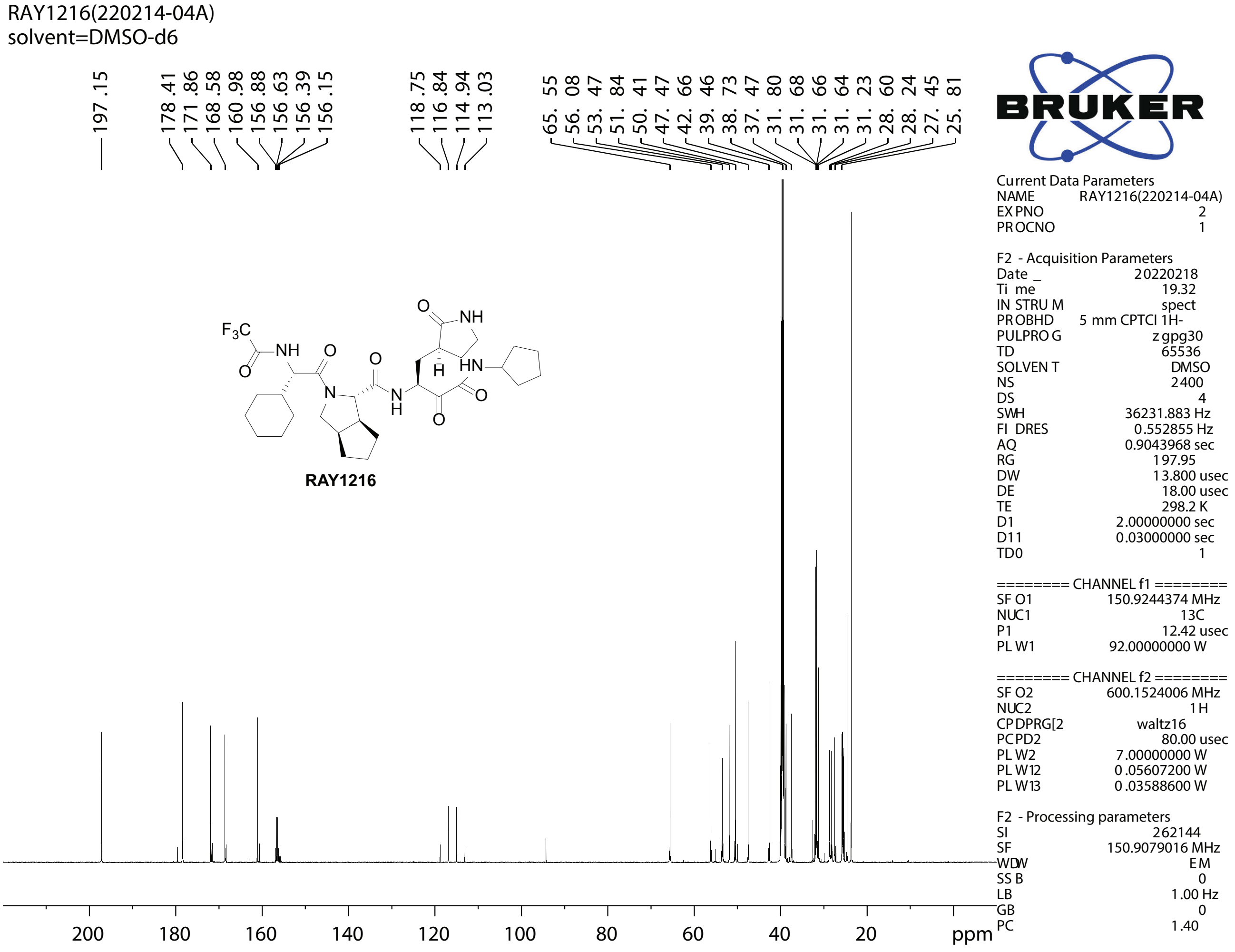

Supplementary Fig. 3 | ^{13}C NMR spectrum of RAY1216 in DMSO-d_6.

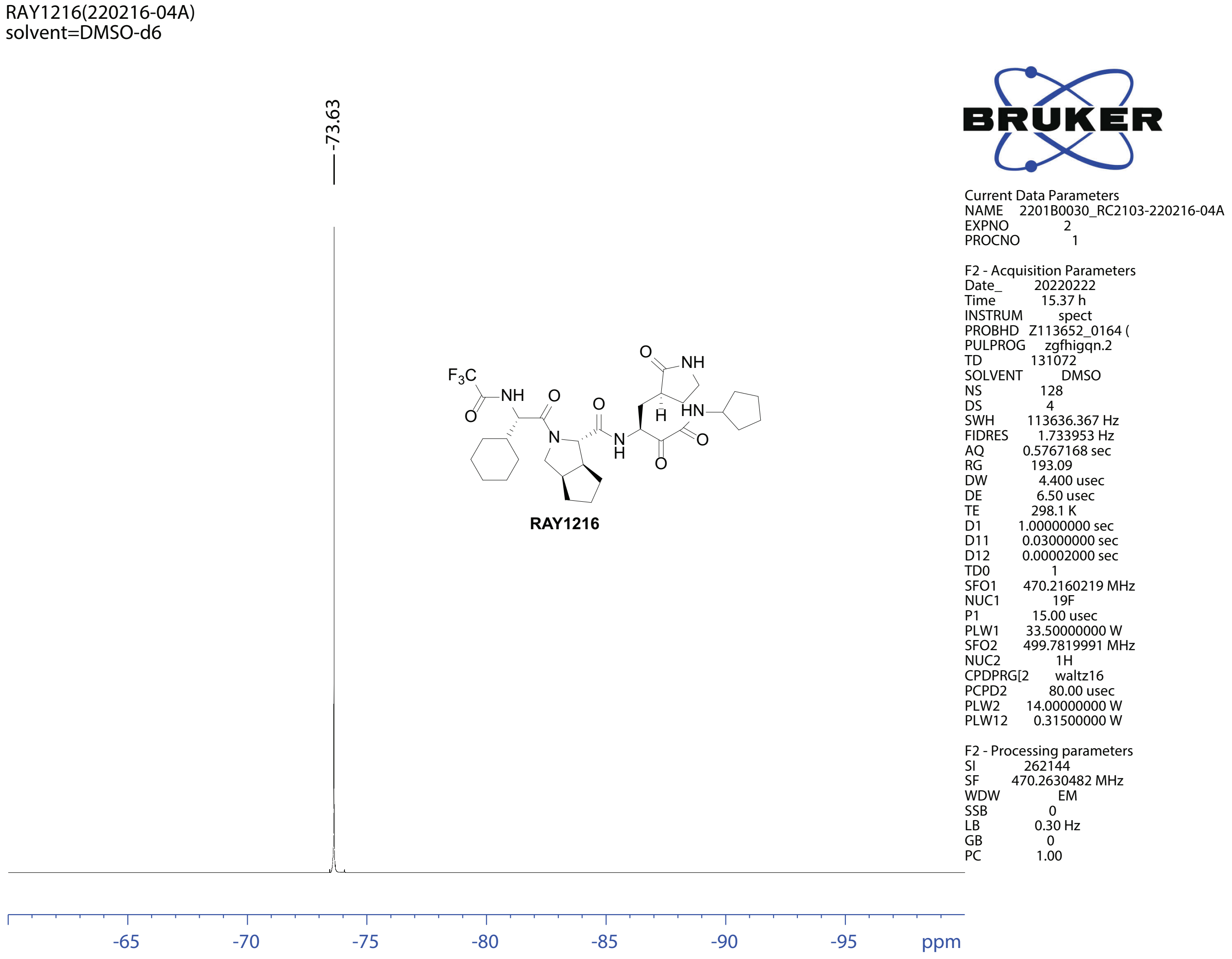

Supplementary Fig. 4 | ^{19}F NMR spectrum of RAY1216 in DMSO-d$_6$.

S1.3. X-ray crystal structure of RAY1216

RAY1216 powder (approximately 30 mg) was dissolved in isopropyl acetate (approximately 600 μL) with gentle stirring until the mixture was saturated. The solution was transferred into a transparent 2 mL MS sample vial after being filtered by a syringe filter. The sample vial was sealed with a parafilm and 5-10 holes on the parafilm were pierced by a syringe needle. The MS vial was placed in a closed brown bottle which contained 0.1 - 0.2 cm level of n-hexane. Solutions were let stand at 20 - 30 °C for 48 hours. After granular white crystals were observed in the MS vial, the isopropyl acetate was removed, and a single crystal of RAY1216 was sealed with silicone grease and subjected to X-ray diffraction on a Rigaku Oxford Diffraction XtaLAB Synergy-S four-circle diffractometer equipped with a CuKα source (λ=1.54184 Å) and a HyPix-6000HE area detector.

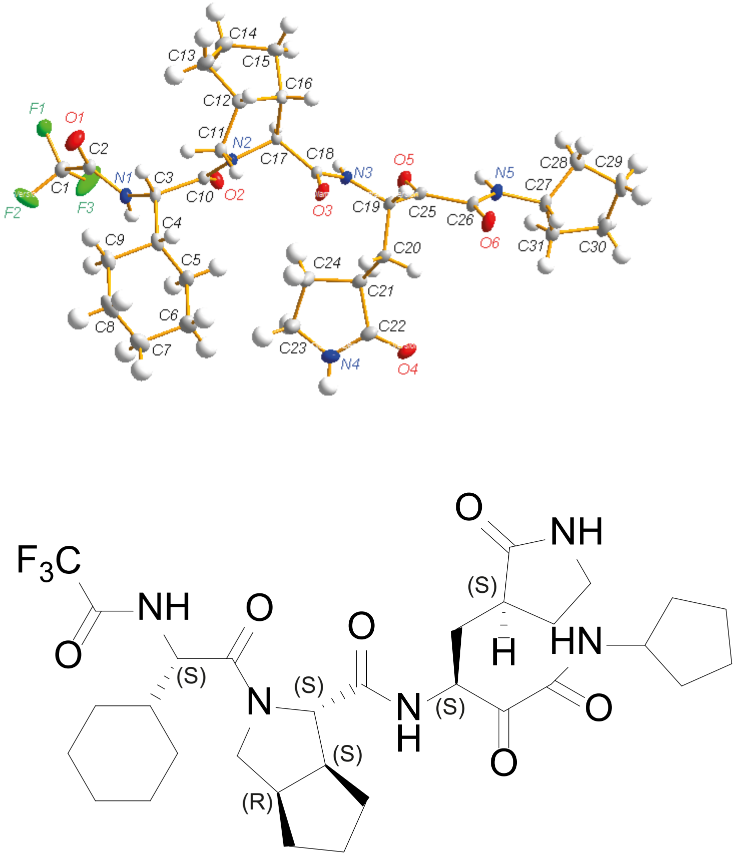

Supplementary Fig. 5 | Single crystal X-ray structure of RAY1216.

Molecule	RAY1216
Radiation Type	CuKα (λ = 1.54184 Å)
Crystal size (mm^3)	0.16 × 0.18 × 0.22
Crystal system	monoclinic
Space group	$P\ 2_1$
**Cell Dimensions**	
a, b, c (Å)	10.6833 9.8921 15.3665
α, β, γ (°)	90.00 91.11 90.00
Cell volume (Å^3)	1623.63 (2)
Cell formula units Z	2
Crystal density$_{calc}$ (g/cm^3)	1.309
Crystal F (000)	680
Absorption coefficient (μ/mm^{-1})	0.862
Index ranges	$-12 \leq h \leq 12$, $-11 \leq k \leq 11$, $-18 \leq l \leq 18$
Cell measurement temperature (K)	149.9 (8)
2θ range for data collection (°)	5.752 to 133.106
Goodness-of-fit on F^2	1.041
Final R indexes [$I \geq 2\sigma(I)$]	R_1 = 0.0351, wR_2 = 0.0914
Final R indexes [all data]	R_1 = 0.0358, wR_2 = 0.0921
Largest diff. peak/hole/e Å^{-3}	0.39/-0.31
Reflections collected/unique	40929/5638 [R_{int} = 0.0391]
Flack parameter	0.10(5)

Supplementary Table 1 | Data collection and statistics of single crystal X-ray structure of RAY1216.

S2. Enzyme kinetics

S2.1. Enzyme activity assay

M^{pro} enzyme assays were performed in enzyme kinetic buffer (20 mM Tris pH 7.8, 150 mM NaCl, 1 mM DTT and 100 µg/mL bovine serum albumin) using Dabcyl-KTSAVLQ/SGFRKME-Edans (Beyotime, #P9733, '/' indicates the M^{pro} cleavage site) as the substrate. The reactions were carried out in 96-well black flat-bottom plates with a final reaction volume of 200 µL. A 10 seconds plate-shaking procedure was used before the data collection. Fluorescent signal by enzyme cleavage of the substrate was monitored on a Molecular Devices FlexStation 3 reader with filters for excitation at 340 nm and emission at 490 nm at 20 °C. The data were recorded by SoftMax Pro 7.0 Software. Minimal data point collection interval time was set to collect as many data points as possible.

S2.2. Molar response coefficient of fluorescent product

M^{pro} at a relatively high concentration (1.0 µM) was assayed with 1.25, 2.5, 5, 10, 20, 40, and 80 µM substrate at 37 °C. Under these experimental conditions the substrate was completely converted into the highly fluorescent product over the course of 20 minutes. The total observed change in fluorescence intensity (ΔF), when plotted against the starting concentration of substrate ([S]), displayed a significant involvement of the frequently seen Dabcyl-EDANS inner-filter effect[1]. The dependence of ΔF on [S] follows a non-linear quadratic function as illustrated in **Supplementary Fig. 6a**. However, within the restricted range of substrate concentrations (1.25 – 20 µM, **Supplementary Fig. 6b**) the dependence of ΔF on [S] is approximately linear, with the slope equal to the differential molar response coefficient of the product r_P = (88.1 ± 3.5) rfu/µM. Thus, in all the following enzyme kinetic analyses we constrained the substrate concentration accordingly ([S] ≤ 20 µM) and treated the molar response coefficient as a fixed parameter, set to the best-fit value of r_P = 88 rfu/µM.

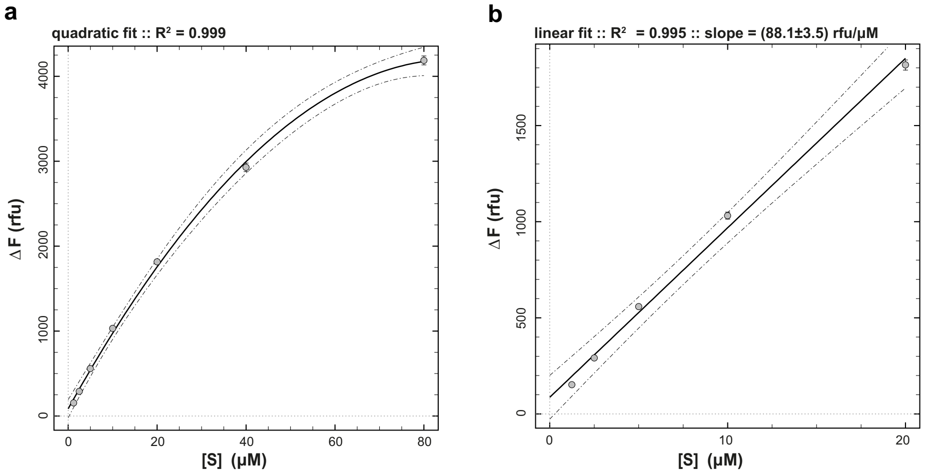

Supplementary Fig. 6 | Determination of molar response coefficient of fluorescent Dabcyl-EDANS cleavage product. a, total observed change in fluorescence intensity (ΔF) upon complete conversion of the substrate to product is plotted against the starting concentration of substrate ([S]) over the complete concentration range (1.25 - 80 µM); data points are fit to the quadratic function. **b**, Fit of the reduced concentration range (1.25 - 20 µM) to a straight line.

S2.3. Determination of the Michaelis constant, K_M

Assays to determine Michaelis constant were conducted using 80 nM M^{pro} and serially diluted substrate (80-1.25 μM, two-fold serial dilution). Reaction progress was monitored for 1 hour and the data points were collected every 9 seconds. Preliminary attempts to determine the Michaelis constant K_M by fitting initial rates vs [S] to the Michaelis-Menten equation were found to be inaccurate due to the Dabcyl-EDANS inner-filter effect[1]. Thus, substrate kinetic parameters were determined by the global fit of reaction progress curves recorded at 1.25, 2.5, 5, 10, and 20 μM to the first-order ordinary differential-equation (ODE) model corresponding to the reaction mechanism shown in ***Scheme S1***, using the software package DynaFit[2,3].

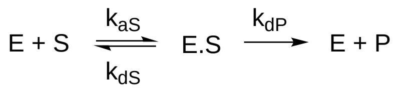

Scheme S1

The mathematical model for the reaction progress curves is auto-generated by DynaFit according to ***Scheme S1*** and is shown as **Eqn (S1)**, where F is the fluorescence intensity at the arbitrary reaction time t; F_0 is the baseline offset or fluorescence intensity observed at time $t = 0$; $r_P = 88$ rfu/μM is the differential molar response of the reaction product; and [P] is the concentration of the reaction product at time t. In its turn, the product concentration is computed by numerically solving the ODE system represented by **Eqns (S2)–(S5)**.

$$F = F_0 + r_P\,[\mathrm{P}] \tag{S1}$$

$$\frac{d[\mathrm{E}]}{dt} = -k_{aS}\,[\mathrm{E}][\mathrm{S}] + k_{dS}\,[\mathrm{E.S}] + k_{dP}\,[\mathrm{E.S}] \tag{S2}$$

$$\frac{d[\mathrm{S}]}{dt} = -k_{aS}\,[\mathrm{E}][\mathrm{S}] + k_{dS}\,[\mathrm{E.S}] \tag{S3}$$

$$\frac{d[\mathrm{E.S}]}{dt} = +k_{aS}\,[\mathrm{E}][\mathrm{S}] - k_{dS}\,[\mathrm{E.S}] - k_{dP}\,[\mathrm{E.S}] \tag{S4}$$

$$\frac{d[\mathrm{P}]}{dt} = +k_{dP}\,[\mathrm{E.S}] \tag{S5}$$

The enzyme-substrate association rate constant k_{aS} was fixed at the diffusion limited constant value of 1.0 $\mu M^{-1}s^{-1}$ (or 1×10^6 $M^{-1}s^{-1}$)[4], whereas the dissociation rate constants k_{dS} and k_{dP} were treated as globally adjustable model parameters. Each individual progress curve was associated with a locally optimized offset parameter F_0. The results of fit for both replicates are illustrated graphically in **Supplementary Fig. 7**.

The best-fit values of adjustable model parameters are listed in **Supplementary Table 2**. The average and standard deviation from replicates ($n = 2$) of the dissociation rate constant is $k_{dS} = (31.0 \pm 0.2)\ s^{-1}$. This particular value of k_{dS} is utilized in subsequent kinetic analyses of M^{pro} inhibition as a fixed model parameter (see below). The corresponding average and standard deviation of the Michaelis constant is $K_M \equiv (k_{dS} + k_{dP})/k_{aS} = (31.0 \pm 0.2)$ μM. This value is identical, within the specified experimental error, to a previously published value of $K_M = (28.2 \pm 3.4)$ μM[5].

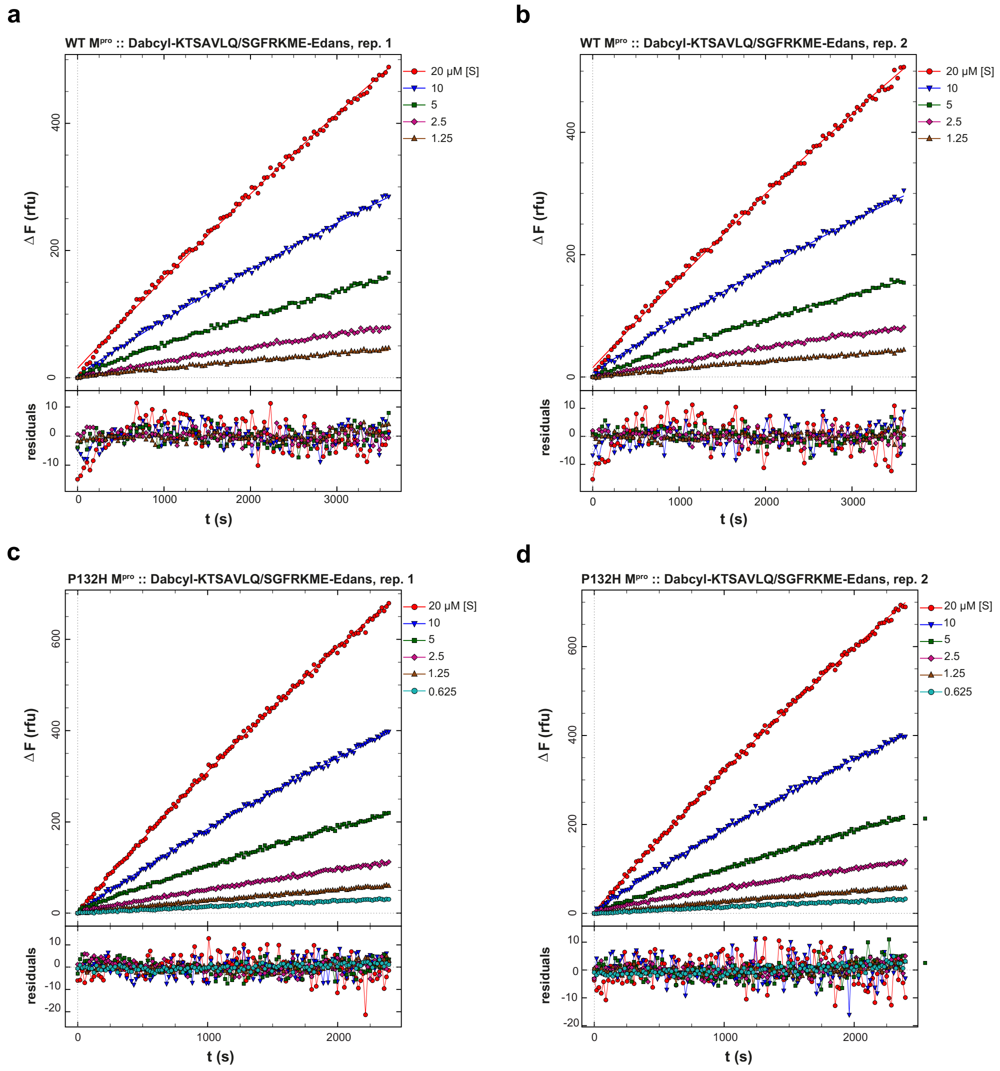

Supplementary Fig. 7 | Substrate-only progress curves of WT M^{pro} and P132H M^{pro} fit to the Michaelis-Menten reaction mechanism shown in *Scheme S1*. The mathematical model for the fitted curves is represented by **Eqns (S1)–(S5)**. For each enzyme, progress curves from 2 replicates are shown.

a

WT M^{pro} parameter	initial	*replicate 1* final	± std.err.	low	high	*replicate 2* final	± std.err.	low	high
k_{dS}, s^{-1}	10	31.1	± 0.8	27.4	35.7	30.8	± 0.8	27.2	35.4
k_{dP}, s^{-1}	0.1	0.0525	± 0.001	0.0481	0.0579	0.0542	± 0.001	0.0497	0.0598

b

P132H M^{pro} parameter	initial	*replicate 1* final	± std.err.	low	high	*replicate 2* final	± std.err.	low	high
k_{dS}, s^{-1}	10	31.6	± 0.5	28.5	35.3	37.3	± 0.7	33.3	42.4
k_{dP}, s^{-1}	0.1	0.1035	± 0.0012	0.0963	0.112	0.12	± 0.0017	0.1103	0.132

Supplementary Table 2 | Results of fits to the substrate-only progress curves using the Michaelis-Menten reaction model. a, Fitting parameters to the WT M^{pro} progress curves; columns labeled as "low" and "high" contain the lower and upper limits, respectively, of non-symmetrical confidence intervals obtained by the profile-*t* method of Bates and Watts[6,7] while using the empirical value of $\Delta SSQ = 5\%$ according to the previously suggested method[8]. **b**, kinetic parameters obtained from fittings to the P132H M^{pro} progress curves.

S2.4. Inhibition kinetics of PF-07321332 and active-site titration

The microscopic rates constants for association and dissociation of PF-07321332, as well as the concentration of M^{pro} active sites hence the enzyme's turnover number k_{cat}, were determined as follows. The enzyme (nominal concentration 80 nM) was assayed at varied concentrations of the inhibitor (maximum 444 nM, 2/3 dilution series down to 17 nM, nine concentrations plus control [I] = 0) in triplicate. 50 µL of 80 µM substrate solution in the assay buffer and 50 µL inhibitor solution were mixed in the reaction plate and the reaction was started by the addition of 100 µL M^{pro} (160 nM wild-type or P132H mutant) in the enzyme kinetics assay buffer using a multichannel pipette. The final concentrations were 20 µM substrate, 1 µM maximum concentration of inhibitor and 80 nM enzyme. The final concentration of DMSO was 0.1% (v/v). Reaction progress was monitored for 1 hour and the data points were collected every 12 seconds. The ten reaction progress curves from each replicate were combined into a global dataset and fit to a differential-equation model corresponding to the reaction mechanism shown in ***Scheme S2*** using the software package DynaFit[2,3].

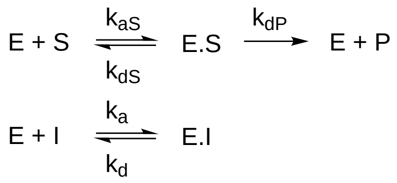

Scheme S2

$$\frac{d[E]}{dt} = -k_{aS}\,[E][S] + k_{dS}\,[E.S] + k_{dP}\,[E.S] - k_{a}\,[E][I] + k_{d}\,[E.I] \tag{S6}$$

$$\frac{d[S]}{dt} = -k_{aS}\,[E][S] + k_{dS}\,[E.S] \tag{S7}$$

$$\frac{d[E.S]}{dt} = +k_{aS}\,[E][S] - k_{dS}\,[E.S] - k_{dP}\,[E.S] \tag{S8}$$

$$\frac{d[P]}{dt} = +k_{dP}\,[E.S] \tag{S9}$$

$$\frac{d[I]}{dt} = -k_{a}\,[E][I] + k_{d}\,[E.I] \tag{S10}$$

$$\frac{d[E.I]}{dt} = +k_{a}\,[E][I] - k_{d}\,[E.I] \tag{S11}$$

To obtain kinetic parameters of PF-07321332 inhibition, the microscopic rate constants k_{aS} and k_{dS} are fixed, whereas the turnover number rate constant $k_{cat} \equiv k_{dP}$ and the active enzyme site concentration are treated as adjustable model parameters (**Supplementary Table 3**). The combined progress curves were fit globally to **Eqn (S1)**, where the product concentration [P] in this case is computed by numerically solving the ODE system represented by **Eqns (S6)–(S11)**.

Thus, the complete list of globally optimized parameters (shared by all progress curves) consists of k_{dP}, k_a, k_d, and [E]; the ten baseline offset values, F_0, each specific to a particular progress curve, were treated as locally adjustable model parameters. The overlay of experimental data on the best-fit model curves and the corresponding plots of instantaneous reaction rates are shown in **Supplementary Fig. 8**. Numerical results are summarized in **Supplementary Table 3**.

The results listed in **Supplementary Table 3** show that all adjustable model parameters were obtained with better than 11% reproducibility. The enzyme was approximately 61% active. The turnover number $k_{cat} = 0.12$ s^{-1} compares well with $k_{cat} = 0.16$ s^{-1} reported previously[5]. Similarly, the inhibition constant $K_i \equiv k_d/k_a = (3.8 \pm 0.2)$ nM determined here compares well with the previously reported value of $K_i = 3.1$ $(1.5 - 6.8)$ nM[9]. The PF-07321332 inhibitor associates relatively rapidly with the enzyme, with bimolecular association rate constant equal to $k_a = 4.9 \times 10^5$ M^{-1}s^{-1}. The drug-target residence time $t_{res} \equiv 1/k_d$ is approximately 9 minutes.

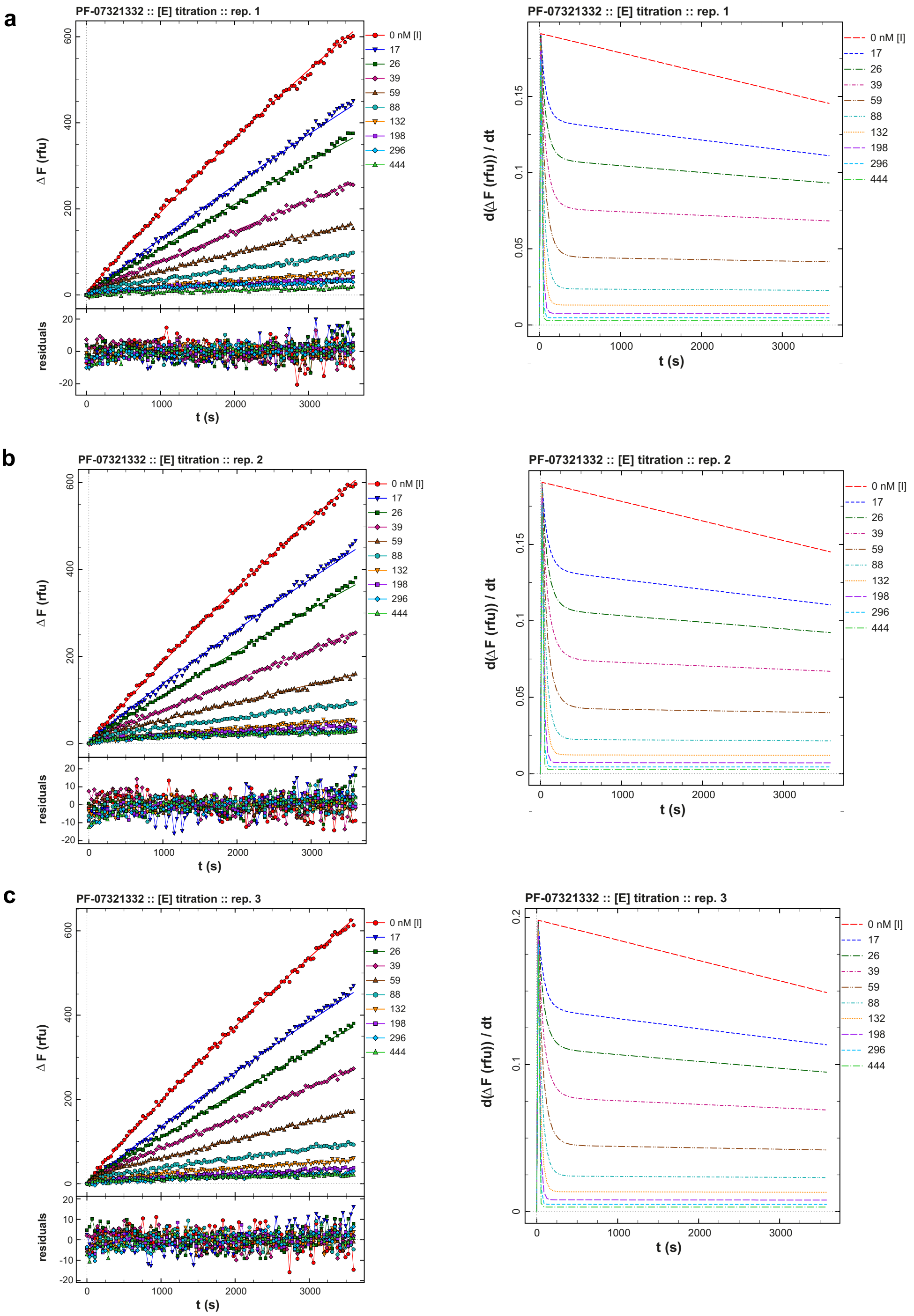

Supplementary Fig. 8 | Inhibition of WT M^{pro} by PF-07321332. *Left panels*: replicated (**a-c**) PF-07321332 inhibition progress curves (dots) are overlaid with best-fit model curves corresponding to the reaction mechanism in ***Scheme S2*** and represented by **Eqns (S6)–(S11)**; the residuals of the fits are shown. *Right panels:* corresponding plots of instantaneous reaction rates for the 3 replicates.

parameter	rep. 1	rep. 2	rep. 3	mean ± stdev	cv(%)	note
k_{aS}, μM^{-1}s^{-1}	1	1	1			fixed
k_{dS}, s^{-1}	31	31	31			fixed
k_{dP}, s^{-1}	0.112	0.112	0.119	0.115 ± 0.004	3.7	
k_a, μM^{-1}s^{-1}	0.53	0.46	0.48	0.49 ± 0.04	7.4	
k_d, s^{-1}	0.0020	0.0016	0.0018	0.0018 ± 0.0002	10.7	
[E], nM	49.5	49.5	48.2	49.1 ± 0.7	1.5	
F_0 1, rfu	7	1.8	3.9			
F_0 2, rfu	-4	3.6	-2.6			
F_0 3, rfu	-3.5	-0.7	-4.7			
F_0 4, rfu	-7.4	-7.6	-2			
F_0 5, rfu	1.1	0.7	5.4			
F_0 6, rfu	4.4	6.1	4.3			
F_0 7, rfu	0.9	3.7	3.7			
F_0 8, rfu	6	9	4.3			
F_0 9, rfu	10.1	8.5	3.5			
F_0 10, rfu	2.8	12.6	7.2			
k_{cat}, s^{-1}	0.112	0.112	0.119	0.115 ± 0.004	3.7	$= k_{dP}$
K_M, μM	31.112	31.112	31.119	31.115 ± 0.004	0.01	$= (k_{dP} + k_{dS})/k_{aS}$
k_{cat}/K_M, M^{-1}s^{-1}	3610	3600	3840	3680 ± 140	3.8	
K_i, nM	3.86	3.60	3.87	3.78 ± 0.16	4.1	$= k_d/k_a$
t_{res}, min	8.2	10.2	9.0	9.1 ± 1.0	10.7	$= 1/k_d$
% enzyme activity	61.9	61.9	60.3	61.3 ± 0.9	1.5	$= 100 \times$ [E]/80.0

Supplementary Table 3 | Results of fit from global analysis of PF-07321332 WT M^{pro} inhibition data. Note that both active enzyme concentration [E] (nominal concentration 80.0 nM) and the turnover number k_{dP} were treated as adjustable parameters. “stdev” is the standard deviation from replicates (n = 3) and "cv(%)" is the corresponding coefficient of variation, cv = 100× stdev/mean. For details see *Methods* section.

S2.5. Inhibition kinetics of RAY1216

The experimental methods of RAY1216 inhibition kinetics were identical to those of PF-07321332. Preliminary analysis of RAY1216 inhibition data revealed that it is possible to reliably determine either the turnover number $k_{cat} \equiv k_{dP}$, or the active enzyme concentration [E], but not both. However, the precise values of these model parameters and in particular the active enzyme concentration strongly influence the best-fit values of the inhibition rate constants k_a and k_d. For this reason, we have analysed the RAY1216 datasets while treating the microscopic rate constant k_{dP} as a fixed parameter determined in the fit of the PF-07321332 data above (the RAY1216 and PF-07321332 experiments were carried out in parallel on the same day using the same M^{pro} preparation).

Thus, the complete list of globally optimized parameters (shared by all progress curves) consists of k_a, k_d, and [E]; the ten baseline offset values, F_0, each specific to a particular progress curve, were again treated as locally adjustable model parameters. The overlay of experimental data on the best-fit model curves and the corresponding plots of instantaneous reaction rates are shown in **Supplementary Fig. 9**. Note that, in comparison with **Supplementary Fig. 8**, the instantaneous rate plots show significantly longer time that is required for near-equilibrium to be established, even at the highest inhibitor concentrations. This graphically illustrates that RAY1216 is a "slow-on, slow-off" inhibitor of M^{pro}, while PF-07321332 is a "fast-on, fast-off" inhibitor. Numerical results of fit are summarized in **Supplementary Table 4**.

The results listed in **Supplementary Table 4** show that all adjustable model parameters were obtained with better than 5% reproducibility. The enzyme was apparently 66% active. This value differs slightly from the 61% enzyme activity determined in the analysis of PF-07321332 data (see **Supplementary Table 3**). We observed that, throughout the working day, the enzymatic activity of M^{pro} decreased slightly but systematically over time. RAY1216 associates relatively slowly with the target enzyme, with bimolecular association rate constant equal to $k_a = 1.8 \times 10^4\ \text{M}^{-1}\text{s}^{-1}$. The inhibition constant of RAY1216, $K_i = 8.4$ nM, is 2.2 times higher than the K_i for PF-07321332 determined here (**Supplementary Table 3**). In contrast, the drug-target residence time is 104/9 = 11.5 times longer.

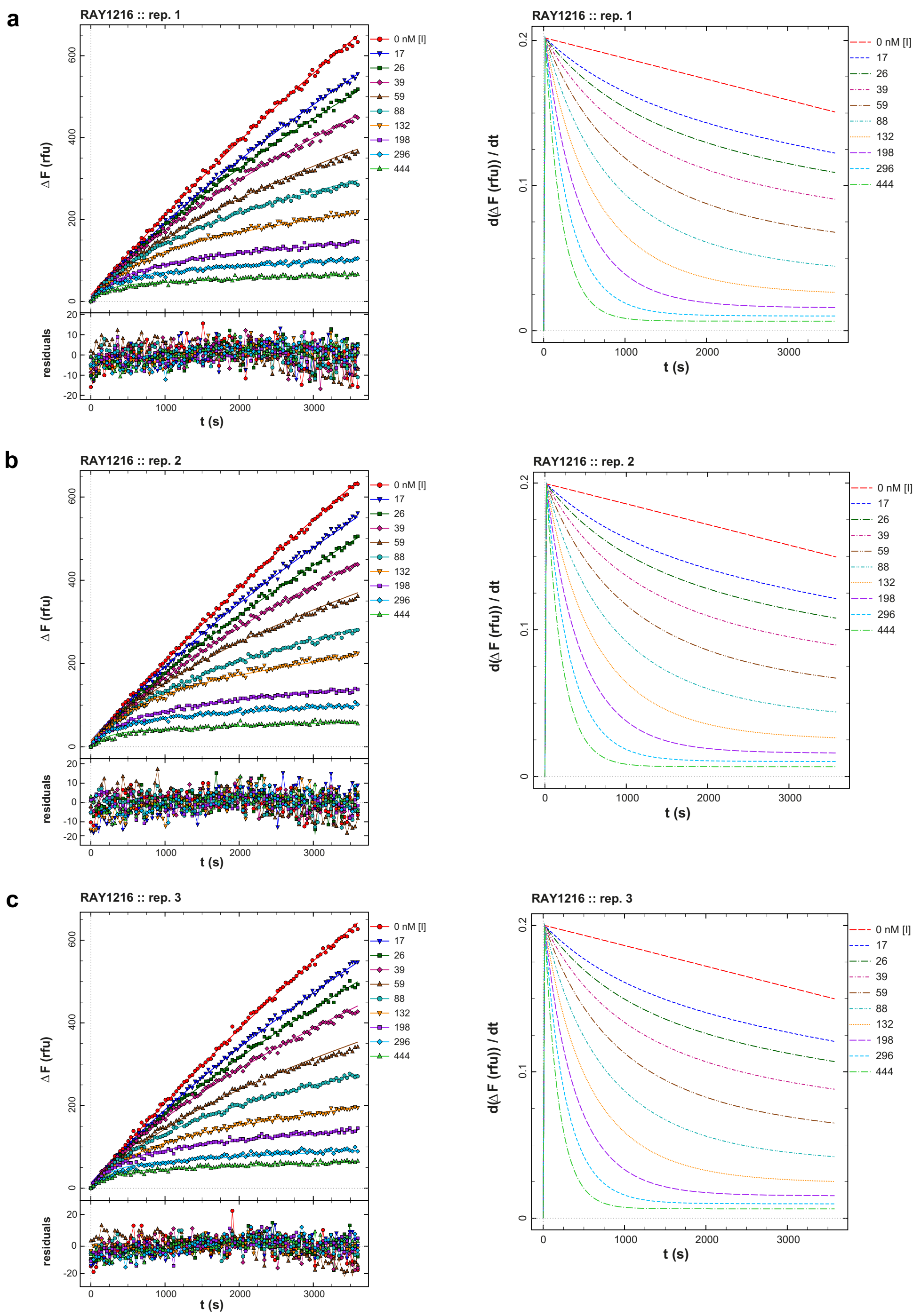

Supplementary Fig. 9 | Inhibition of WT M^{pro} by RAY1216. *Left panels*: replicated (**a-c**) RAY1216 inhibition progress curves (dots) are overlaid with best-fit model curves corresponding to the reaction mechanism in ***Scheme S2*** and represented by **Eqns** (**S6**)–(**S11**); the residuals of the fits are shown. *Right panels:* corresponding plots of instantaneous reaction rates for the 3 replicates.

parameter	rep. 1	rep. 2	rep. 3	mean ± stdev	cv(%)	note
k_{aS}, μM^{-1}s^{-1}	1	1	1			fixed
k_{dS}, s^{-1}	31	31	31			fixed
k_{dP}, s^{-1}	0.11	0.11	0.11			fixed
k_a, μM^{-1}s^{-1}	0.0183	0.0187	0.0208	0.0193 ± 0.0014	7.1	
k_d, s^{-1}	0.000153	0.000161	0.000169	0.000161 ± 0.000008	4.9	
[E], nM	53.3	52.7	52.8	52.9 ± 0.3	0.6	
F_0 1, rfu	15.9	10.3	12.7			
F_0 2, rfu	11.1	14.7	12.0			
F_0 3, rfu	10.2	5.3	7.9			
F_0 4, rfu	8.5	2.6	8.7			
F_0 5, rfu	-2.0	2.0	-3.5			
F_0 6, rfu	5.4	-0.9	3.3			
F_0 7, rfu	7.2	13.9	2.8			
F_0 8, rfu	1.1	-1.4	9.4			
F_0 9, rfu	4.7	5.1	4.6			
F_0 10, rfu	0.7	-3.2	5.1			
K_i, nM	8.4	8.6	8.1	8.4 ± 0.2	2.9	$= k_d/k_a$
t_{res}, min	109	104	99	104 ± 5	4.9	$= 1/k_d$
% enzyme activity	66.6	65.9	66.0	66.2 ± 0.4	0.6	$= 100 \times [E]/80.0$

Supplementary Table 4 | Results of fit from global analysis of RAY1216 wild-type M^{pro} inhibition data. The active enzyme concentration [E] (nominal concentration 80.0 nM) is being treated as adjustable parameter while the turnover number k_{dP} is held fixed. “stdev” is the standard deviation from replicates (n = 3) and "cv(%)" is the corresponding coefficient of variation, cv = 100× stdev/mean. For details see *Methods* section.

S2.6. Inhibition of the P132H mutant

The P132H mutant of M^{pro} was assayed under experimental conditions that were identical to those utilized for the assay of the wild-type enzyme, except for the fact that the maximum RAY1216 concentration was 1000 nM as opposed to 444 nM for PF-0732332. The results of fit are summarized graphically in the Main Manuscript *Extended Data Fig. 9*; the numerical results are listed in **Supplementary Table 5**.

	parameter	rep. 1	rep. 2	rep. 3	mean ± stdev	cv(%)	note
**PF-07321332**	k_{aS}, μM^{-1}s^{-1}	1	1	1			fixed
	k_{dS}, s^{-1}	35	35	35			fixed
	k_{dP}, s^{-1}	0.1607	0.1885	0.1924	0.1805 ± 0.0173	9.6	
	k_a, μM^{-1}s^{-1}	1.46	1.33	1.00	1.26 ± 0.237	18.8	
	k_d, s^{-1}	0.0077	0.0065	0.0047	0.0063 ± 0.0015	24.4	
	[E], nM	44.3	38.6	39.2	40.7 ± 3.1	7.6	
	k_{cat}, s^{-1}	0.1607	0.1885	0.1924	0.1805 ± 0.0173	9.6	$= k_{dP}$
	K_M, μM	35.161	35.189	35.192	35.2 ± 0.02	0	$= (k_{dP}+k_{dS})/k_{aS}$
	k_{cat}/K_M, M^{-1}s^{-1}	4570	5357	5467	5131 ± 489	9.5	
	K_i, nM	5.27	4.89	4.66	4.9 ± 0.3	6.3	$= k_d/k_a$
	t_{res}, min	2.2	2.6	3.6	3 ± 1	26.3	$= 1/k_d$
	% activity	55.3	48.3	49	50.9 ± 3.9	7.6	= 100 × [E] / 80
**RAY1216**	k_{aS}, μM^{-1}s^{-1}	1	1	1			fixed
	k_{dS}, s^{-1}	35	35	35			fixed
	k_{dP}, s^{-1}	0.147	0.2043	0.2083	0.1865 ± 0.0343	18.4	
	k_a, μM^{-1}s^{-1}	0.02386	0.02881	0.02615	0.02627 ± 0.002477	9.4	
	k_d, s^{-1}	0.000199	0.000244	0.000222	0.000222 ± 0.000024	10.1	
	[E], nM	63.5	50.6	48.3	54.1 ± 8.2	15.2	
	K_i, nM	8.34	8.46	8.5	8.4 ± 0.1	1	$= k_d/k_a$
	t_{res}, min	83.8	68.4	75	76 ± 8	10.2	$= 1/k_d$
	% activity	79.4	63.1	60.4	67.7 ± 10.3	15.2	= 100 × [E] / 80

Supplementary Table 5 | Kinetic parameters of P132H M^{pro} inhibition by PF-07321332 and RAY1216.

S3. M^{pro} crystal structures

S3.1. Simulated-annealing 2mFo-DFc composite omit map densities

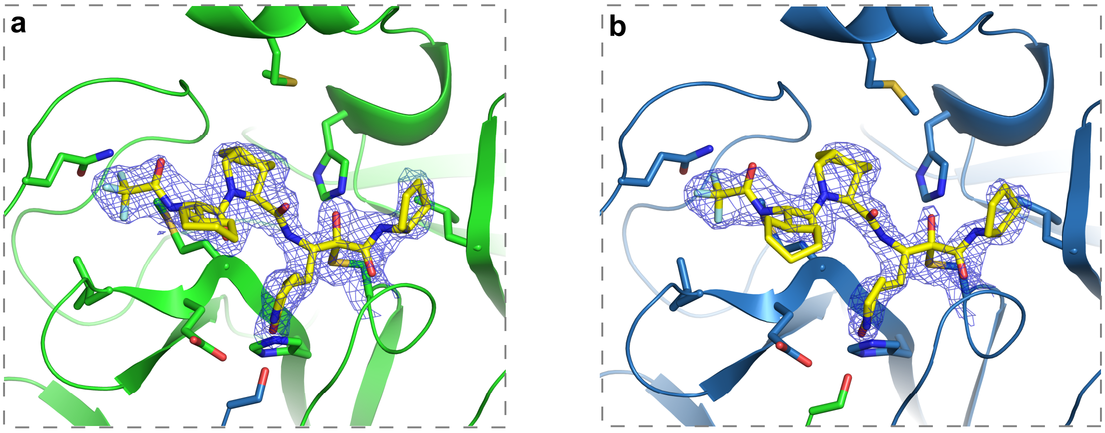

Supplementary Fig. 10 | Simulated-annealing 2mFo-DFc composite omit map densities showing bound RAY1216 and covalent linkages between RAY1216 and Cys145. The simulated-annealing composite omit map for the M^{pro} dimer was calculated by omitting bound RAY1216 and Cys145 in both monomers. Shown densities are contoured at 1.1σ, models are colored according to Fig. 3. **a**, density in protomer A. **b**, density in protomer B.

S3.2. Comparison of active site structures

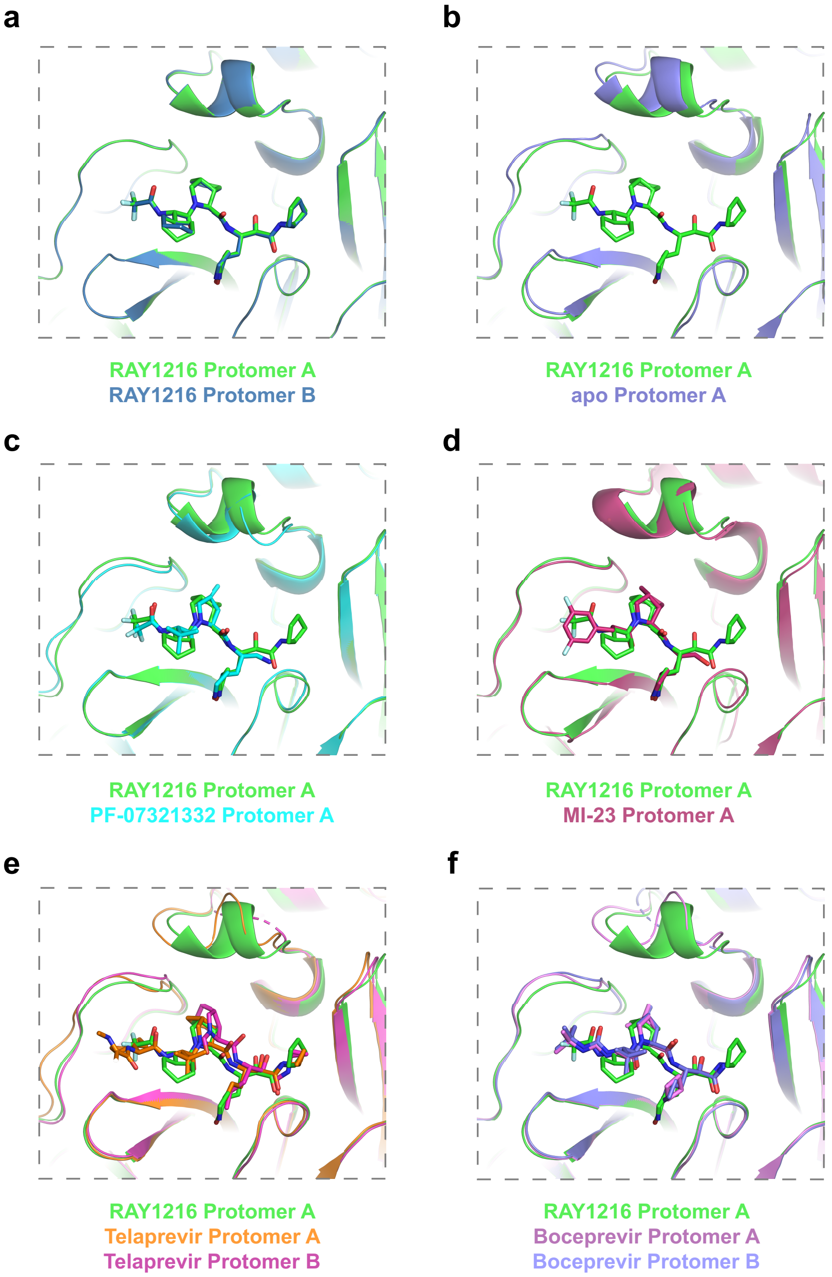

Supplementary Fig. 11 | Comparison of active site structures of M^{pro} in different inhibitor complexes shows active site structural plasticity. Structures of boceprevir (PDB: 7com[10]), MI-23 (PDB: 7d3i[10]), PF-07321332 (PDB: 7rfw[9]), telaprevir (PDB: 7c7p[10]) in complex with M^{pro} are compared with RAY1216:M^{pro} structures.

S3.3. Protein crystallographic refinement statistics data

	Parameter	**M^{pro} free enzyme PDB: 8IGO**	**M^{pro} + RAY1216 PDB: 8IGN**
Data collection	wavelength (Å)	0.97849	0.97853
	Space group	*C* 2	*P* 1
	**Cell Dimensions**		
	a, b, c (Å)	97.39 82.49 51.42	55.83 60.94 64.04
	α, β, γ (°)	90.00 115.52 90.00	80.03 68.34 70.67
	Resolution (Å)	43.46-2.00 (2.05-2.00)	59.42-2.00 (2.05-2.00)
	R_{merge}	0.031 (0.327)	0.067 (0.504)
	No. Reflections (total)	156563 (11225)	168870 (11201)
	No. Reflections (unique)	23876 (1817)	48627 (3545)
	$I/\sigma I$	31.6 (5.1)	9.9 (2.1)
	Completeness (%)	96.2 (98.8)	97.5 (96)
	Multiplicity	6.6 (6.2)	3.5 (3.2)
Refinement	No. Reflections	23851 (2089)	47154 (4676)
	R_{work}/R_{free}	0.2059/0.2491	0.1862/0.2312
	**Number of atoms**		
	Protein	2314	4654
	Inhibitor	-	90
	Water	100	255
	**B-Factors**		
	Protein	43.06	38.84
	Inhibitor	-	37.25
	Water	44.64	45.39
	**R.m.s. deviations**		
	Bond lengths (Å)	0.008	0.0066
	Bond angles (°)	0.87	1.478
Validation	MolProbity score	1.41	1.94
	Clashscore	4.15	6.02
	Poor rotamers (%)	0	2.13
	**Ramchandran plot**		
	Favored (%)	96.66	94.82
	Allowed (%)	3.34	5.18
	Disallowed (%)	0	0

Supplementary Table 6 | Data collection statistics of M^{pro} crystal structures.

S4. Antiviral activity of RAY1216

S4.1. Cytotoxicity of RAY1216 in Vero E6 cells

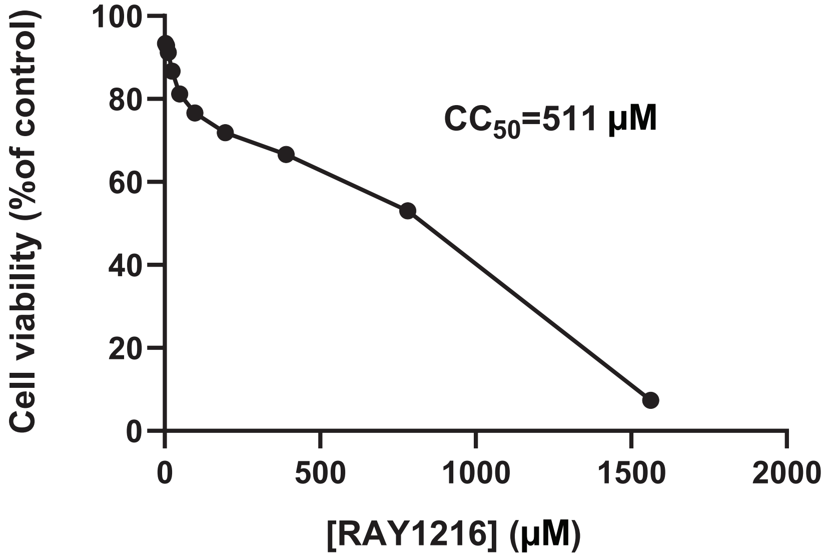

Supplementary Fig. 12 | Cytotoxicity of RAY1216 in Vero E6 cells.

S4.2. Antiviral titres of RAY1216 and PF-07321332 in Vero E6 cells

	**RAY1216** (nM)				**PF-07321332** (nM)			
	Plaque-reduction		CPE-inhibition		Plaque-reduction		CPE-inhibition	
SARS-CoV-2 virus strain	EC_{50}	EC_{90}	EC_{50}	EC_{90}	EC_{50}	EC_{90}	EC_{50}	EC_{90}
WT	116	320	90	228	62	142	90	191
Alpha	80	200	121	351	50	115	69	205
Beta	88	259	262	688	62	146	138	327
Delta	69	212	92	254	40	105	69	177
Omicron BA.1	81	169	78	208	50	89	100	214
Omicron BA.5	91	293	149	363	61	144	105	270
Omicron XBB.1.9.1	135	417	159	415	49	138	136	420

Supplementary Table 7 | Antiviral titres of RAY1216 and PF-07321332 in Vero E6 cells as assessed by plaque reduction or cytopathic effect (CPE) inhibition assay.

S5. Pharmacokinetics

S5.1. Pharmacokinetics of RAY1216 in K18-hACE2 transgenic mice

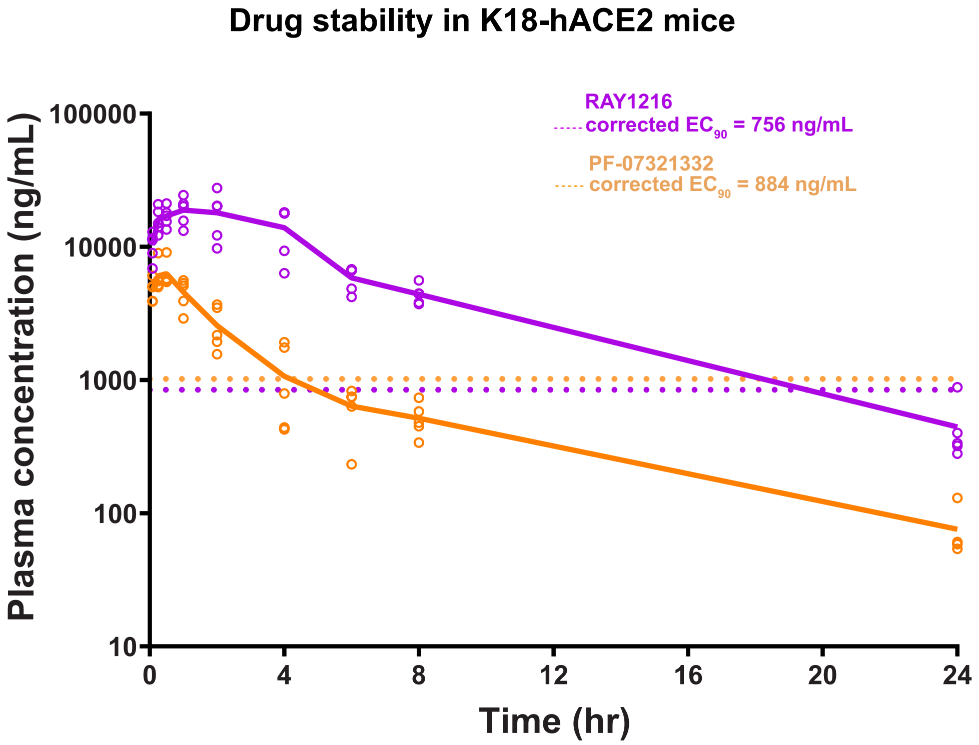

Supplementary Fig. 13 | 24-hour plasma concentrations of RAY1216 and PF-07321332 in K18-hACE2 transgenic mice after being gavaged with 600 mg/kg/day for 5 days. 5 mice are tested for each drug, average plasma concentrations are connected by lines. Dotted lines indicate EC_{90} values (against Delta variant, by CPE-inhibition method) corrected for plasma protein binding.

	RAY1216	PF-07321332
$AUC_{(0-t)}$ (h·ng/mL)	140000 ± 34000	23000 ± 4200
$T_{1/2}$ (hr)	4.8 ± 1.0	5.3 ± 1.7
Mouse plasma protein bound drug / Free drug	0.785 / 0.215	0.9 / 0.1
Plasma concentration at 24 hr (ng/mL)	443 ± 248	76 ± 36
EC_{90} against Delta variant by CPE inhibition (ng/mL)	162.5	88.4
EC_{90} corrected for plasma protein binding (ng/mL)	756	884
Plasma concentration at 24 hr / corrected EC_{90}	0.586	0.086

Supplementary Table 8 | Pharmacokinetics parameters of RAY1216 and PF-07321332 in K18-hACE2 mice.

References

1. Liu, Y. *et al.* Use of a fluorescence plate reader for measuring kinetic parameters with inner filter effect correction. *Anal. Biochem.* **267**, 331–5 (1999). URL http://doi.org/10.1006/abio.1998.3014.
2. Kuzmič, P. Program DYNAFIT for the analysis of enzyme kinetic data: Application to HIV proteinase. *Anal. Biochem.* **237**, 260–273 (1996). URL http://doi.org/10.1006/abio.1996.0238.
3. Kuzmič, P. DynaFit - A software package for enzymology. *Meth. Enzymol.* **467**, 247–280 (2009). URL http://doi.org/10.1016/S0076-6879(09)67010-5.
4. Fersht, A. *Structure and Mechanism in Protein Science: A Guide to Enzyme Catalysis and Protein Folding* 3rd edn (W. H. Freeman, New York, 1999).
5. Ma, C. *et al.* Boceprevir, GC-376, and calpain inhibitors II, XII inhibit SARS-CoV-2 viral replication by targeting the viral main protease. *Cell Res.* **30**, 678–692 (2020). URL http://doi.org/10.1038/s41422-020-0356-z.
6. Bates, D. M. & Watts, D. G. *Nonlinear Regression Analysis and its Applications* (Wiley, New York, 1988).
7. Watts, D. G. Parameter estimation from nonlinear models. *Meth. Enzymol.* **240**, 24–36 (1994). URL http://doi.org/10.1016/S0076-6879(94)40041-5.
8. Johnson, K. A. Fitting enzyme kinetic data with KinTek Global Kinetic Explorer. *Meth. Enzymol.* **467**, 601–626 (2009). URL http://doi.org/10.1016/S0076-6879(09)67023-3.
9. Owen, D. R. *et al.* An oral SARS-CoV-2 M^{pro} inhibitor clinical candidate for the treatment of COVID-19. *Science* **374**, 1586–1593 (2021). URL http://doi.org/10.1126/science.abl4784.
10. Qiao, J. *et al.* SARS-CoV-2 M^{pro} inhibitors with antiviral activity in a transgenic mouse model. *Science* **371**, 1374–1378 (2021). URL http://doi.org/10.1126/science.abf1611.
