# Supplementary material for: Preclinical evaluation of the SARS-CoV-2 Mpro inhibitor RAY1216 shows improved pharmacokinetics compared with nirmatrelvir
Source: Nat Microbiol. 2024 Mar 29;9(4):1075–88. doi: 10.1038/s41564-024-01618-9 (PMC10994847; doi:10.1038/s41564-024-01618-9)
Supplement: Supplementary file 2 — Reporting Summary [file 41564_2024_1618_MOESM2_ESM.pdf]

nature portfolio

Corresponding author(s): Xiaoli Xiong, Zifeng Yang, Nanshan Zhong, Shu-Hui Chen

Last updated by author(s): Jan 20, 2024

Reporting Summary

Nature Portfolio wishes to improve the reproducibility of the work that we publish. This form provides structure for consistency and transparency in reporting. For further information on Nature Portfolio policies, see our Editorial Policies and the Editorial Policy Checklist.

Statistics

For all statistical analyses, confirm that the following items are present in the figure legend, table legend, main text, or Methods section.

n/a	Confirmed	
☐	☒	The exact sample size (*n*) for each experimental group/condition, given as a discrete number and unit of measurement
☐	☒	A statement on whether measurements were taken from distinct samples or whether the same sample was measured repeatedly
☐	☒	The statistical test(s) used AND whether they are one- or two-sided<br>*Only common tests should be described solely by name; describe more complex techniques in the Methods section.*
☒	☐	A description of all covariates tested
☒	☐	A description of any assumptions or corrections, such as tests of normality and adjustment for multiple comparisons
☐	☒	A full description of the statistical parameters including central tendency (e.g. means) or other basic estimates (e.g. regression coefficient) AND variation (e.g. standard deviation) or associated estimates of uncertainty (e.g. confidence intervals)
☐	☒	For null hypothesis testing, the test statistic (e.g. *F*, *t*, *r*) with confidence intervals, effect sizes, degrees of freedom and *P* value noted<br>*Give P values as exact values whenever suitable.*
☒	☐	For Bayesian analysis, information on the choice of priors and Markov chain Monte Carlo settings
☒	☐	For hierarchical and complex designs, identification of the appropriate level for tests and full reporting of outcomes
☒	☐	Estimates of effect sizes (e.g. Cohen's *d*, Pearson's *r*), indicating how they were calculated

Our web collection on statistics for biologists contains articles on many of the points above.

Software and code

Policy information about availability of computer code

Data collection	Molecular device SoftMax Pro 7.0 Software was used to measure enzyme kinetics data and luminescence in the replicon assay. ABI PRISM 7500 real-time PCR System (Applied Biosystems) was used to measure RNA levels in the virus inhibition assay.
Data analysis	DynaFit 4, XDS software (BUILT 20220220), CCP4 7.1.018, Phaser MR 2.8.3, Coot 0.9.6, Refmac 5.8.0267, Phoenix WinNonlin software (version 8.2.0), SHELXT, SHELXL. IBM SPSS Statistics Version 25.0. GraphPad Prism version 8.0

For manuscripts utilizing custom algorithms or software that are central to the research but not yet described in published literature, software must be made available to editors and reviewers. We strongly encourage code deposition in a community repository (e.g. GitHub). See the Nature Portfolio guidelines for submitting code & software for further information.

Data

Policy information about availability of data

All manuscripts must include a data availability statement. This statement should provide the following information, where applicable:

- Accession codes, unique identifiers, or web links for publicly available datasets
- A description of any restrictions on data availability
- For clinical datasets or third party data, please ensure that the statement adheres to our policy

The single crystal X-ray structure of RAY1216 has been deposited in The Cambridge Crystallographic Data Centre (www.ccdc.cam.ac.uk ,CCDC) with the CDS Entry number DIDVEV and CCDC number 2251675 (DOI: 10.5517/ccdc.csd.cc2fl1ps). The data can be obtained free of charge from CCDC via www.ccdc.cam.ac.uk/

data_request/cif. The coordinates and structure factors of Mpro crystal structures have been deposited in the Protein Data Bank (www.wwpdb.org) under accession numbers 8IGO (Apo Mpro) and 8IGN (RAY1216:Mpro). Source data are provided with this paper.

Human research participants

Policy information about studies involving human research participants and Sex and Gender in Research.

Reporting on sex and gender	Not applicable.
Population characteristics	Not applicable.
Recruitment	Not applicable.
Ethics oversight	Not applicable.

Note that full information on the approval of the study protocol must also be provided in the manuscript.

Field-specific reporting

Please select the one below that is the best fit for your research. If you are not sure, read the appropriate sections before making your selection.

☒ Life sciences ☐ Behavioural & social sciences ☐ Ecological, evolutionary & environmental sciences

For a reference copy of the document with all sections, see nature.com/documents/nr-reporting-summary-flat.pdf

Life sciences study design

All studies must disclose on these points even when the disclosure is negative.

Sample size	The sample sizes were similar to those reported in previous publications (https://doi.org/10.1038/s41586-020-2312-y and https://doi.org/10.1126/science.abf1611).For mouse model using in the antiviral study, there were seven mice in each group (six groups in total). For the ICR mouse model in vivo pharmacokinetic study, there were five male mice and five female mice per group in different sets of experiments. For the SD rat model in vivo pharmacokinetic study, there were five male rats and five female rats per group in different sets of experiments. For the K18-hACE2 mouse model in vivo pharmacokinetic study, there were five K18-hACE2 female mice per group in different sets of experiments. This is to meet the requirement for statistical analysis while ensuring good technical reproducibility. Other assays were performed for duplicates or three replicates, which were also sufficient for a good statistical analysis.
Data exclusions	No data were excluded from the analyses presented in the manuscript.
Replication	As for the plasma concentration, viral titers, and HE stain experiments, at least 3 animals for each group (at each detecting time point) were tested, and some of them were tested in two or four replications. All attempts at replication were successful. The replicates of the enzyme kinetic assay and the experiments based on the cells were noted in the figure legends and methods.
Randomization	For the antiviral study, we randomly divided 42 K18-hACE2 transgenic mice into six groups. For lung tissues analyzed with histological staining and virus titer determination assays, we chose a specific number of mice for examinations, and histological images were selected randomly from the corresponding experimental groups. For the pharmacokinetic study in vivo, there were no randomizations in dividing ICR mice, SD rats, and K18-hACE2 mice into different groups.
Blinding	The investigators were not blinded to allocation during experiments andoutcome assessment. The relevant quantitative experiments in the manuscript, such as the determination of virus titer, viral gene level expression, drug concentrations in plasma, and the records the changes of the animals' weight and survival rate, need to be correctly and clearly labeled on the tubes or cages. All samples are tested and analyzed in accordance with the protocol, and the results would not be effected by the subjective judgment of the investigators. The evaluation of histopathological changes required a qualified and experienced pathologist to observe all samples. The results of the comprehensive evaluation of all samples in different groups were described in the manuscript to exclude personal subjective bias.

Reporting for specific materials, systems and methods

We require information from authors about some types of materials, experimental systems and methods used in many studies. Here, indicate whether each material, system or method listed is relevant to your study. If you are not sure if a list item applies to your research, read the appropriate section before selecting a response.

Materials & experimental systems

n/a	Involved in the study
☒	☐ Antibodies
☐	☒ Eukaryotic cell lines
☒	☐ Palaeontology and archaeology
☐	☒ Animals and other organisms
☒	☐ Clinical data
☒	☐ Dual use research of concern

Methods

n/a	Involved in the study
☒	☐ ChIP-seq
☒	☐ Flow cytometry
☒	☐ MRI-based neuroimaging

Eukaryotic cell lines

Policy information about cell lines and Sex and Gender in Research

Cell line source(s)	Vero E6 cells: ATCC, CRL-1586; HEK 293T cells: ATCC,CRL-3216.
Authentication	All cell lines were frequently checked for the cellular morphologies, growth rates and functions in our lab and were not commonly misidentified.
Mycoplasma contamination	The cell lines were not contaminated by mycoplasma as determined by using the Lonza Mycoplasma Detection Kit.
Commonly misidentified lines (See ICLAC register)	No commonly misidentified cell lines were used.

Animals and other research organisms

Policy information about studies involving animals; ARRIVE guidelines recommended for reporting animal research, and Sex and Gender in Research

Laboratory animals	For the SARS-CoV-2 animal experiment, the five to six-weeks old female K18-hACE2-transgenic C57BL/6 mice were provided by Gempharmatech Co., Ltd. (Jiangsu, China). The animals were fed every day with the fodder purchased from Beijing Keao Xieli Feed Co., Ltd. and the general quality standards, hygienic standards and conventional nutritional ingredient index requirements in feeds are tested in accordance with GB14924.2-2001 and GB14924.3-2010 standards. All work with live SARS-CoV-2 was conducted in the Biosafety Level 3 (BLS3) Laboratories. The mice were randomly divided into six groups (7 mice per group). All mice were kept in SPF (specific pathogen free) facilities.<br>For the pharmacokinetic in vivo study, the four to six-weeks old ICR mice and the six to eight-weeks old SD rats were provided by Vital River Laboratory Animal Technology Co., Ltd. (Beijing, China). The six-weeks old K18-hACE2-transgenic C57BL/6 mice were provided by Gempharmatech Co., Ltd. (Jiangsu, China). The animals were fed every day with the fodder purchased from Wuhan WQJX Bio-Technology Co., Ltd. All the animals were house in controlled temperature (20-26°C), humidity (40-70%) and lighting conditions (12 h light/ 12 h dark cycles).
Wild animals	The study did not involve wild animals.
Reporting on sex	All the K18-hACE2-transgenic C57BL/6 mice were female. All the ICR mice and SD rats for the pharmacokinetics study were half male and half female.
Field-collected samples	No sample is collected from the field.
Ethics oversight	The antiviral studies were approved by the Guangzhou Medical University Ethics Committee of Animal Experiments (IACUC certificate No.: GZL0008). The PK studies were approved by the ethics committee the Institute Animal Care and Use Committee (IACUC) of Precedo Pharmaceuticals Co., Ltd. The IACUC No. for rat PK studies and mouse (including ICR mouse and K18-hACE2 mouse) PK studies are IACUC-20230303-2 and IACUC-20230303-3, respectively. All plasmas used in the RAY1216 plasma stability experiment are from commercially sources. The plasmas of CD-1 mouse and SD rat were purchased from Vital River Laboratories (Beijing, China). The cynomolgus monkey plasma was purchased from Xishan Zhongke Laboratory Animal Co., Ltd (Suzhou, China) and the plasmas of beagle dog (#CAN00PLK2Y2N) and human (#HUMANPLK2P2N) were purchased from BioIVT (NY, USA).

Note that full information on the approval of the study protocol must also be provided in the manuscript.
