# Supplementary material for: Preclinical evaluation of the SARS-CoV-2 Mpro inhibitor RAY1216 shows improved pharmacokinetics compared with nirmatrelvir
Source: Nat Microbiol. 2024 Mar 29;9(4):1075–88. doi: 10.1038/s41564-024-01618-9 (PMC10994847; doi:10.1038/s41564-024-01618-9)
Supplement: Supplementary file 4 — 1H NMR. [file 41564_2024_1618_MOESM4_ESM.pdf]

RAY1216(220214-04A)
solvent=DMSO-d6

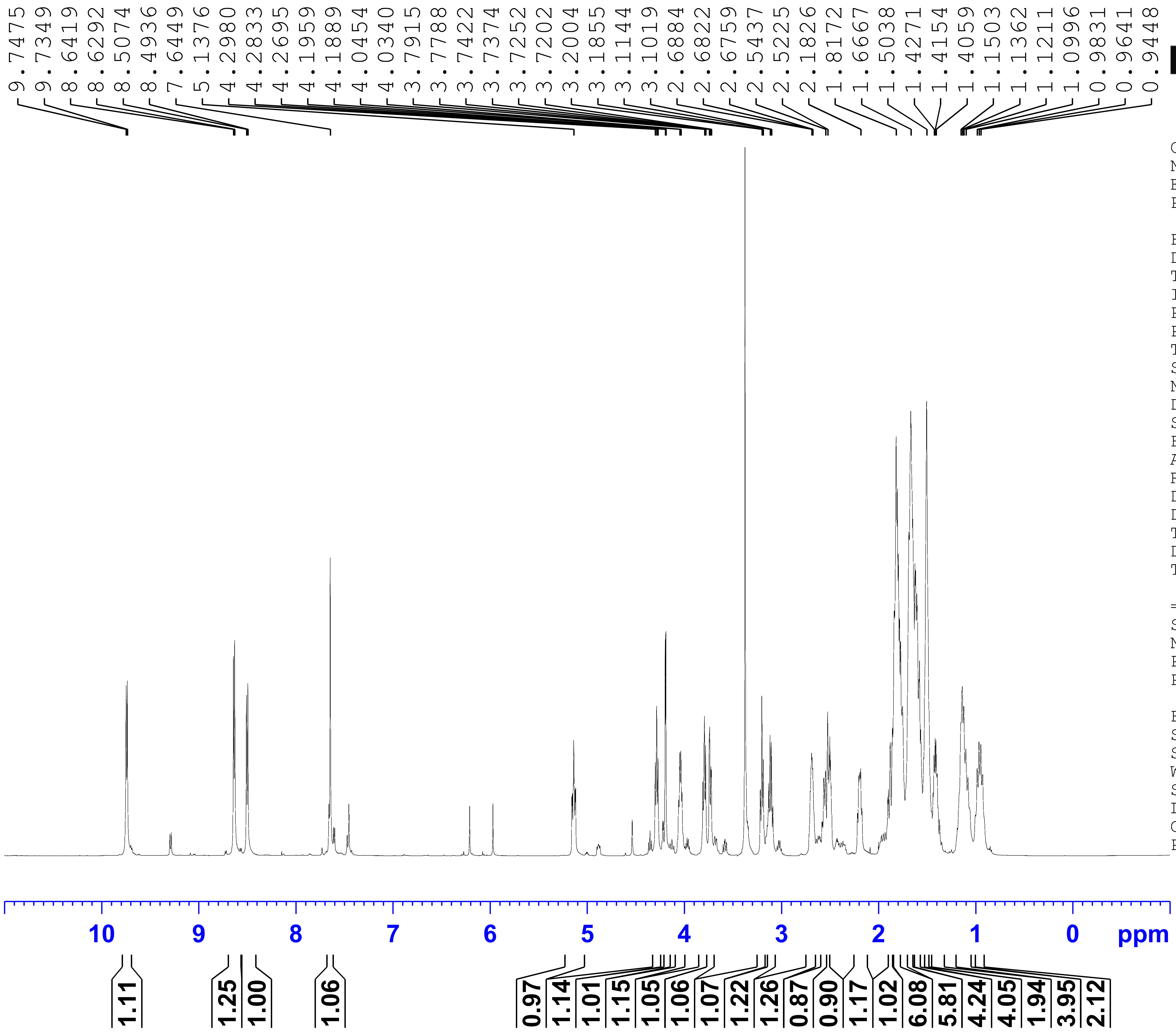

Current Data Parameters
NAME RAY1216(220214-04A)_1022456
EXPNO 102
PROCNO 1

F2 - Acquisition Parameters
Date_ 20220218
Time_ 17.32
INSTRUM spect
PROBHD 5 mm CPTCI 1H-
PULPROG zg30
TD 65536
SOLVENT DMSO
NS 16
DS 2
SWH 9615.385 Hz
FIDRES 0.146719 Hz
AQ 3.4078720 sec
RG 4.52
DW 52.000 usec
DE 10.00 usec
TE 298.1 K
D1 1.00000000 sec
TD0 1

======== CHANNEL f1 ========
SFO1 600.1540810 MHz
NUC1 1H
P1 7.16 usec
PLW1 7.00000000 W

F2 - Processing parameters
SI 65536
SF 600.1499930 MHz
WDW EM
SSB 0
LB 0.30 Hz
GB 0
PC 1.00
