# Supplementary material for: Preclinical evaluation of the SARS-CoV-2 Mpro inhibitor RAY1216 shows improved pharmacokinetics compared with nirmatrelvir
Source: Nat Microbiol. 2024 Mar 29;9(4):1075–88. doi: 10.1038/s41564-024-01618-9 (PMC10994847; doi:10.1038/s41564-024-01618-9)
Supplement: Supplementary file 5 — 13C NMR. [file 41564_2024_1618_MOESM5_ESM.pdf]

RAY1216(220214-04A)
solvent=DMSO-d6

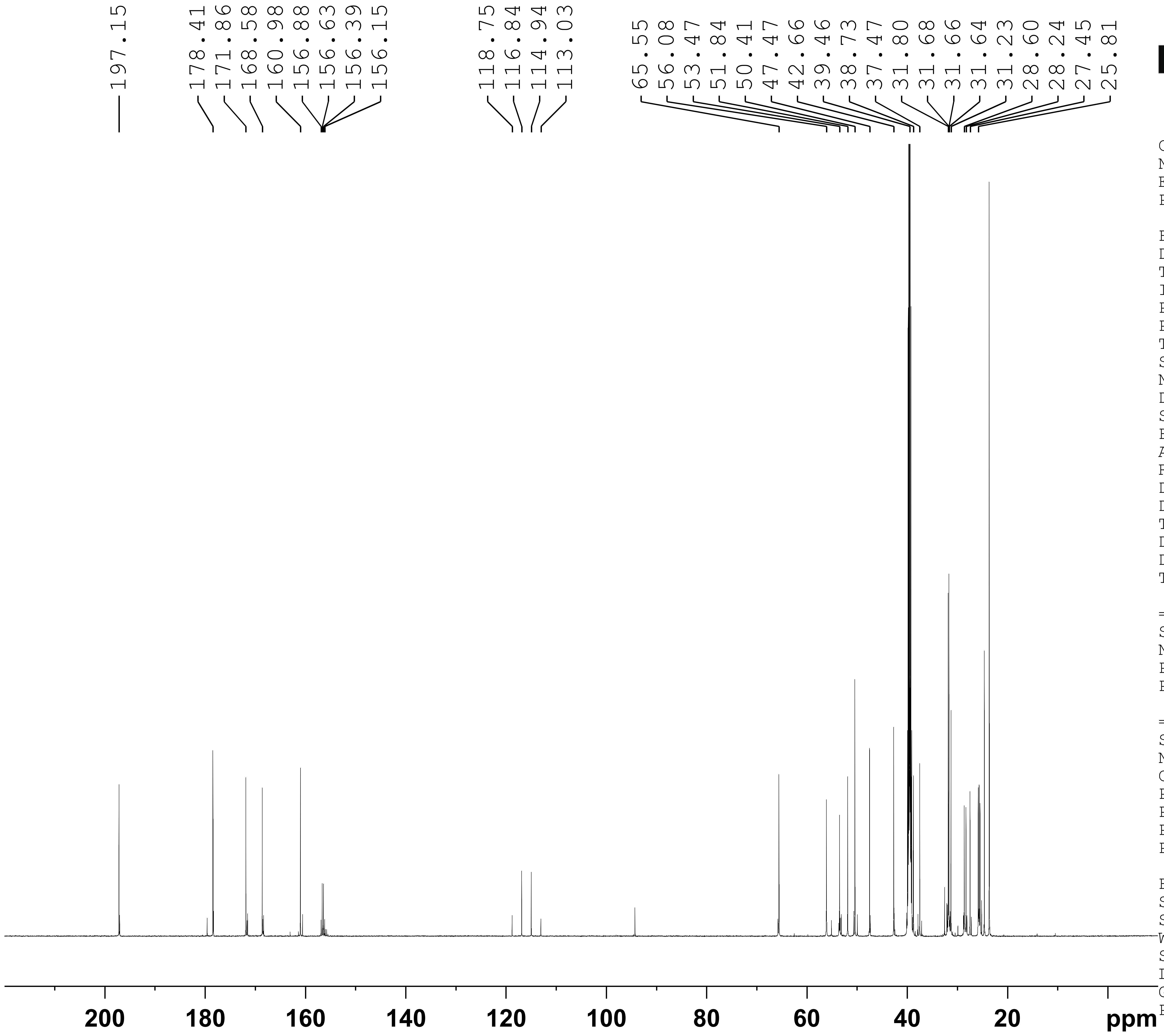

Current Data Parameters
NAME RAY1216(220214-04A)
EXPNO 2
PROCNO 1

F2 - Acquisition Parameters

Date_	20220218	
Time	19.32	
INSTRUM	spect	
PROBHD	5 mm CPTCI 1H-	
PULPROG	zgpg30	
TD	65536	
SOLVENT	DMSO	
NS	2400	
DS	4	
SWH	36231.883	Hz
FIDRES	0.552855	Hz
AQ	0.9043968	sec
RG	197.95	
DW	13.800	usec
DE	18.00	usec
TE	298.2	K
D1	2.00000000	sec
D11	0.03000000	sec
TD0	1	

======== CHANNEL f1 ========

SFO1	150.9244374	MHz
NUC1	13C	
P1	12.42	usec
PLW1	92.00000000	W

======== CHANNEL f2 ========

SFO2	600.1524006	MHz
NUC2	1H	
CPDPRG[2	waltz16	
PCPD2	80.00	usec
PLW2	7.00000000	W
PLW12	0.05607200	W
PLW13	0.03588600	W

F2 - Processing parameters

SI	262144	
SF	150.9079016	MHz
WDW	EM	
SSB	0	
LB	1.00	Hz
GB	0	
PC	1.40	
