# Supplementary material for: Preclinical evaluation of the SARS-CoV-2 Mpro inhibitor RAY1216 shows improved pharmacokinetics compared with nirmatrelvir
Source: Nat Microbiol. 2024 Mar 29;9(4):1075–88. doi: 10.1038/s41564-024-01618-9 (PMC10994847; doi:10.1038/s41564-024-01618-9)
Supplement: Supplementary file 6 — 19F NMR. [file 41564_2024_1618_MOESM6_ESM.pdf]

RAY1216(220216-04A)
solvent=DMSO-d6

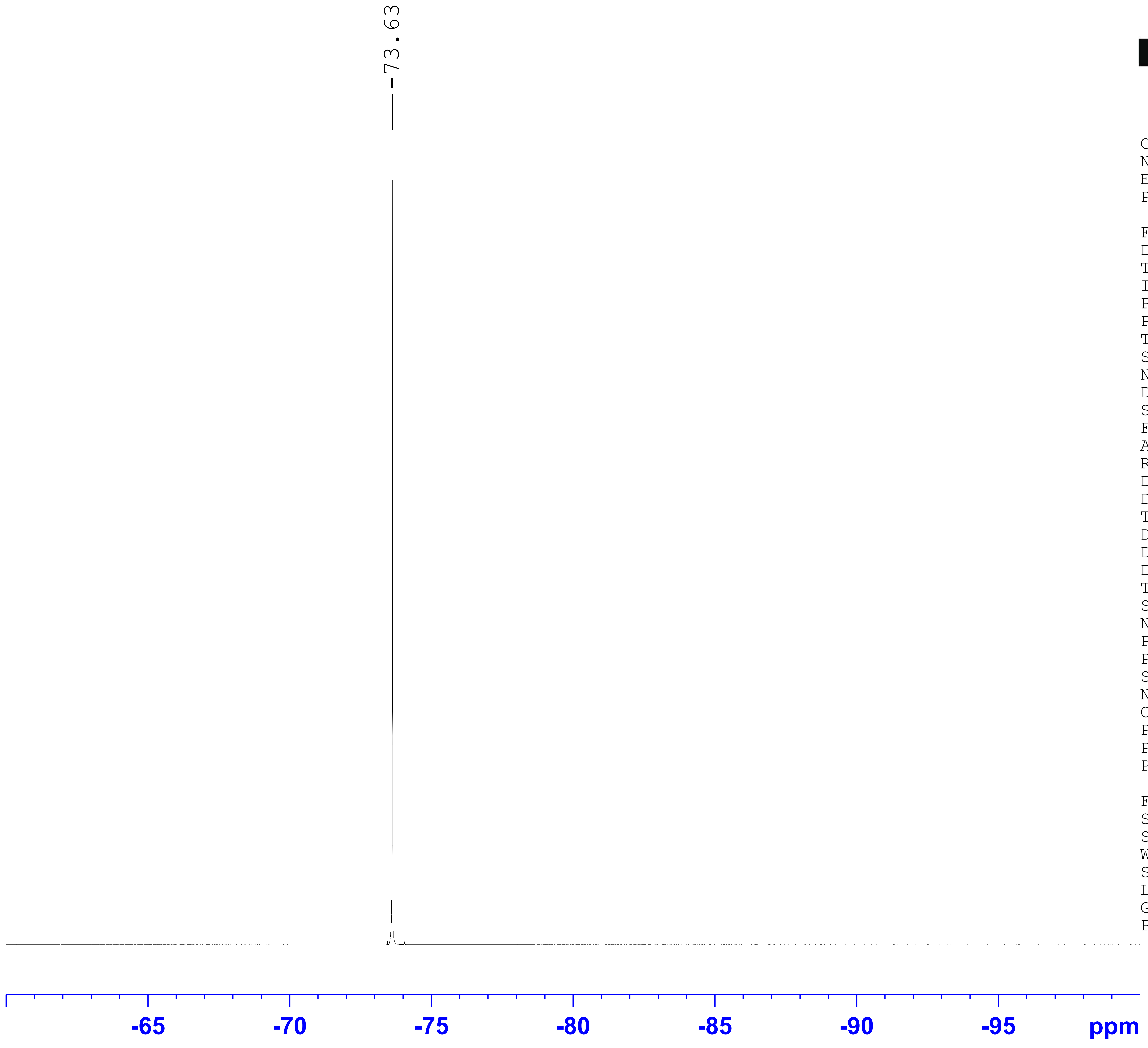

Current Data Parameters		
NAME	2201B0030_RC2103-220216-04A	
EXPNO	2	
PROCNO	1	
F2 - Acquisition Parameters		
Date_	20220222	
Time_	15.37	h
INSTRUM	spect	
PROBHD	Z113652_0164 (	
PULPROG	zgfhigqn.2	
TD	131072	
SOLVENT	DMSO	
NS	128	
DS	4	
SWH	113636.367	Hz
FIDRES	1.733953	Hz
AQ	0.5767168	sec
RG	193.09	
DW	4.400	usec
DE	6.50	usec
TE	298.1	K
D1	1.00000000	sec
D11	0.03000000	sec
D12	0.00002000	sec
TD0	1	
SFO1	470.2160219	MHz
NUC1	19F	
P1	15.00	usec
PLW1	33.50000000	W
SFO2	499.7819991	MHz
NUC2	1H	
CPDPRG[2	waltz16	
PCPD2	80.00	usec
PLW2	14.00000000	W
PLW12	0.31500000	W
F2 - Processing parameters		
SI	262144	
SF	470.2630482	MHz
WDW	EM	
SSB	0	
LB	0.30	Hz
GB	0	
PC	1.00	
